# An Intelligent Disassembly Sequence Optimisation Framework for End-of-Life EV Batteries Using Adaptive Operator Selection

**DOI:** 10.3390/biomimetics11090631

**Published:** 2026-09-04

**Authors:** Jun Huang, Mengying He, Guanghui Yang, Xiuyi Ao, Yupin Zhang, Natalia Hartono, Duc T. Pham

**Affiliations:** 1 School of Information Engineering, Wuhan University of Technology, Wuhan 430070, China; 315051@whut.edu.cn; 2 School of Mechanical and Electronic Engineering, Wuhan University of Technology, Wuhan 430070, China; ygh2025202452@whut.edu.cn; 3 Wuhan Power Battery Recycling Technology Co., Ltd., Wuhan 430014, China; aoxiuyi@gem.com.cn (X.A.); zhangyuping@gem.com.cn (Y.Z.); 4 Exeter Digital Enterprise Systems (ExDES) Laboratory, Engineering Department, University of Exeter, Exeter EX4 4QF, UK; n.hartono@exeter.ac.uk; 5 Department of Mechanical Engineering, School of Engineering, University of Birmingham, Birmingham B15 2TT, UK; d.t.pham@bham.ac.uk

**Keywords:** end-of-life EV battery, disassembly sequence optimisation, Bees Algorithm, reinforcement learning, adaptive operator selection, human–robot collaborative disassembly

## Abstract

End-of-life (EoL) electric vehicle (EV) batteries comprise numerous interconnected components with complex topological and precedence relationships. These constraints significantly increase the difficulty of disassembly sequence planning (DSP), as feasible sequences must satisfy multiple dependency requirements. Moreover, the large number of possible disassembly alternatives creates a vast search space, making sequence optimisation susceptible to combinatorial explosion and convergence to local optima. Therefore, effective DSP requires both robust constraint-handling mechanisms to ensure sequence feasibility and efficient optimisation strategies to identify high-quality solutions. To address these challenges, this paper proposes a disassembly sequence optimisation method that integrates a hard-constraint rule base, the linear upper confidence bound (LinUCB) algorithm, and the Bees Algorithm (BA). First, a disassembly-oriented hard-constraint rule base is developed to standardise the identification of component topological relationships and precedence constraints, thereby ensuring the generation of feasible disassembly sequences. A LinUCB-based contextual adaptive operator-selection mechanism is subsequently introduced to dynamically select neighbourhood operators according to the current search state. A weighted multi-criteria evaluation function incorporating disassembly time, payment cost, and human–robot utility is integrated into the BA. Two representative EoL-EV battery case studies with different levels of structural complexity are used for validation. Across 50 independent runs, LinUCB-BA reduced the mean normalised weighted objective value by 39.01% and 28.12% relative to simplified swarm optimisation (SSO) and teaching–learning-based optimisation (TLBO), respectively, in the 27-component case, and by 7.19% and 2.33% in the 16-component case. Compared with the enhanced discrete Bees Algorithm (EDBA) ablation baseline, further reductions of 1.98% and 0.43% were achieved, together with lower run-to-run variability. These results indicate that the proposed framework is effective for the two investigated battery disassembly scenarios, while broader validation across additional battery architectures and operating conditions remains necessary.

## 1. Introduction

Electric vehicles (EVs) are widely regarded as an important means of reducing carbon emissions from the transport sector [1]. Consequently, many governments have introduced policies and incentives to accelerate their manufacture and adoption [2]. As EV deployment continues to expand, the first generation of traction batteries has begun to reach end of life (EoL), with projections suggesting that several million battery packs will be retired annually by 2030 [3]. These retired batteries contain valuable materials, including lithium, cobalt, and nickel, making their recovery both economically attractive and environmentally important.

The recovery of these materials depends heavily on efficient battery disassembly [4,5]. However, EV battery packs are complex multi-level assemblies comprising cells, modules, and pack-level structures connected through various fastening approaches, including screwing, welding, and adhesive bonding [6,7]. Accessing a target component often requires the prior removal of multiple other components, creating intricate interdependencies throughout the disassembly process. As a result, determining how a battery should be dismantled is a challenging planning task.

Disassembly sequence planning (DSP) aims to determine the order of component removal to improve disassembly efficiency, safety, and recovery performance [8]. For EoL-EV batteries, DSP is particularly difficult because feasible disassembly operations must satisfy numerous engineering constraints, including geometric interference, disassembly directions, tool requirements, and precedence relationships between components [8,9]. At the same time, the number of possible disassembly sequences increases rapidly as battery complexity grows [9]. DSP therefore becomes a large-scale combinatorial optimisation problem in which solution quality must be balanced against strict requirements for physical feasibility.

A variety of optimisation approaches have been applied to DSP. Exact methods, such as integer linear programming (ILP), can identify optimal solutions but suffer from computational costs that increase exponentially with problem size, limiting their applicability to realistic battery packs [10]. Metaheuristic methods offer greater scalability and have consequently become popular for complex disassembly problems. However, two persistent difficulties remain. First, engineering constraints are often represented incompletely or enforced through penalty mechanisms, allowing physically infeasible solutions to emerge during the search. Second, the search process itself may converge prematurely, causing the optimisation to become trapped in local optima. Effective DSP therefore requires both rigorous constraint handling and robust global search capabilities.

The challenge of constraint handling is particularly important for EoL-EV batteries because disassembly decisions depend on multiple forms of heterogeneous information, including topological relationships, disassembly precedence, process requirements, historical disassembly knowledge, and geometric interference among components. Although knowledge graph techniques have been used to organise such information effectively [11], incorporating it directly into the optimisation process remains difficult. Consequently, a significant challenge is to develop a representation that can encode these constraints in a form that both guarantees feasibility and can be efficiently utilised during sequence generation.

Even when constraints are represented effectively, the optimisation process must still navigate a large and complex search space. To address this challenge, a range of intelligent search strategies has been investigated. The Bees Algorithm (BA) [12], inspired by the foraging behaviour of honeybees, has been applied successfully to disassembly optimisation problems. More recently, adaptive operator-selection strategies based on multi-armed bandits [13] and reinforcement learning have attracted attention because they enable the search process to adjust its behaviour according to the current optimisation state. By balancing exploration of under-utilised operators against exploitation of successful ones, these methods can improve search efficiency and solution quality. Recent studies have combined reinforcement learning with the BA for DSP [14] and with particle swarm optimisation for power-battery disassembly [15].

While these developments demonstrate the value of adaptive search strategies, existing studies have focused primarily on improving optimisation performance. Comparatively less attention has been devoted to embedding process-level disassembly constraints directly into the generation of candidate solutions [11,13,14,15]. Consequently, optimisation algorithms may still expend considerable computational effort evaluating infeasible sequences that violate physical or procedural requirements. This limitation highlights the need for an approach that integrates robust constraint handling with adaptive optimisation within a unified framework.

To address this need, the present study proposes a disassembly sequence optimisation method that integrates a process-oriented hard-constraint rule base, the linear upper confidence bound (LinUCB) algorithm, and the BA. The hard-constraint rule base incorporates topological relationships, process constraints, and component precedence rules into the physical interference matrix, thereby resulting in an enhanced interference matrix and restricting the search process to feasible regions of the solution space. Based on this constraint-guided framework, LinUCB is introduced to adaptively select search operators according to the current optimisation state, balancing exploration and exploitation. The selected operators are embedded into the BA framework to enhance search efficiency and maintain solution diversity. Through the integration of constraint handling and adaptive operator selection, the proposed method aims to generate high-quality disassembly sequences that satisfy complex engineering constraints and achieve superior optimisation performance.

The main contributions of this work are threefold:

- A process-oriented hard-constraint rule base that generates an improved interference matrix and ensures the feasibility of initial disassembly solutions for EoL-EV batteries.
- A LinUCB-based adaptive operator-selection mechanism that dynamically guides the search process within the BA framework.
- An integrated DSP approach that combines process-level hard constraints, adaptive operator selection, and BA optimisation within a unified framework, and is validated through EoL-EV battery case studies.

The remainder of the paper is organised as follows: Section 2 provides a brief literature review of disassembly constraint modelling methods and DSP for EoL-EV batteries. Section 3 describes the proposed method. Section 4 presents case studies validating the approach. Section 5 discusses the advantages and limitations of the method. Finally, Section 6 concludes the paper, summarises the main findings of the study and proposes directions for future research.

## 2. Related Work

### 2.1. Disassembly Constraint Modelling Methods

Disassembly modelling is the essential first step in planning the disassembly sequence. Its main purpose is to describe the key features of the disassembly target formally, in line with established process rules and boundary constraints. These features include component structural characteristics, inter-component joining relationships, spatial geometric interference conditions, and disassembly precedence constraints. Disassembly modelling methods can be classified into three main types: matrix-based, graph-theory-based, and Petri net-based.

Matrix-based methods, the most established and widely adopted foundation, quantify inter-component constraints through two-dimensional or multi-dimensional matrices. Disassembly constraints were modelled using combined interference, contact, and connection matrices, supplemented by a subassembly instability matrix, with binary encoding to simplify inter-component relationships [16]. A spatial interference matrix for Liquid Crystal Display (LCD) televisions was established to derive feasible disassembly sequences [17].

Graph-theory-based methods use a “node-edge” structure to represent the logical disassembly constraints between components, typically through directed graphs and AND/OR graphs [18]. A knowledge graph was constructed to capture dynamic disassembly relations and component dependencies, with topological sorting and backtracking used to derive optimal disassembly sequences [19]. In a related approach, subassemblies were encoded as hyperedges within a hypergraph, and the resulting model was converted into an AND/OR graph for disassembly planning and validated through two realistic case studies [20]. AND/OR graphs have also been applied to parallel disassembly, representing product structures and disassembly systems in a form that is straightforward to implement and well suited to parallel and targeted disassembly planning [21].

Petri nets are a graphical and formal modelling tool for discrete event systems, consisting of places, transitions, arcs, and tokens [22]. A Petri net-based method was proposed to address the disassembly and recycling of Waste Electrical and Electronic Equipment (WEEE), exploiting its ability to model the disassembly processes dynamically [23].

### 2.2. Disassembly Sequence Planning for EoL-EV Batteries

Building on these models, disassembly sequence planning (DSP) determines the order in which components are removed and is central to the economical and safe recycling of EoL-EV batteries. Human–robot collaborative disassembly combines the adaptability of human operators with the precision of robots to improve efficiency [21], for which safe physical interaction between human and robot is a core requirement [24]. DSP methods are commonly grouped into three categories: exact algorithms, metaheuristic algorithms, and hybridisation with machine learning or deep learning techniques. Exact methods such as ILP were initially used to model disassembly sequence planning problems [10]. Although this approach could guarantee theoretical optimality, its computational complexity increased exponentially with the number of components [10]. To address this challenge, metaheuristic methods were adopted, using iterative search processes to approach a globally optimal solution without exhaustively exploring all possibilities, and have proved efficient on large-scale, high-dimensional disassembly problems [8]. Several metaheuristic methods have been applied to EV battery and related disassembly problems. A hybrid particle swarm optimisation with Q-learning was developed for the human–robot collaborative disassembly of power batteries, with a self-learning module selecting among perturbation and local-search operators at each iteration to minimise completion time [15]. A multi-objective improved genetic algorithm was tested across 22 benchmark cases [25], a multi-objective evolutionary algorithm was proposed for collaborative human–robot disassembly sequence planning [26], and a self-adaptive simplified swarm optimisation (SSO) was applied to the disassembly sequencing problem [27].

The BA, which mimics the foraging behaviour of honeybees, has been applied to a wide range of complex optimisation problems [12] and features prominently in disassembly research. Fibonacci sequences were integrated into the standard BA to improve solution quality with fewer parameter settings [28]. A time-energy bi-objective model was developed for sequence-dependent disassembly under uncertainty, solved with an improved BA combined with stochastic programming [8]. The BA was also applied to the DSP of gear pumps [29], and a Pareto-based enhanced discrete BA was proposed and validated on a simplified computer chassis, outperforming three competing algorithms across several test cases [30]. Task allocation and sequence planning for human–robot collaborative disassembly have likewise been addressed using BA [31].

While standard metaheuristics excel at global exploration, mathematical refinements adopted to improve constraint handling. Bandit-based adaptive operator selection, in which each operator is treated as an arm and chosen according to its recent reward, has been studied in evolutionary computation for over a decade, including dynamic upper confidence bound schemes [13] and UCB-based selection within the Multi-Objective Evolutionary Algorithm Based on Decomposition (MOEA/D) [32]. Reinforcement learning has also been embedded in metaheuristics: a hybrid BA with reinforcement learning was proposed for disassembly sequence planning [14], while related learning and evolutionary approaches include a multi-objective evolutionary algorithm for human–robot collaborative DSP [26] and a multi-agent reinforcement-learning framework with Q-learning for adapting disassembly task planning to environmental changes [33]. For multi-objective disassembly, an improved multi-objective hybrid Grey Wolf Optimiser was developed [34], whilst a human–cyber–physical framework for an Industry 5.0 collaboration cell optimised disassembly sequence and task allocation together, evaluating task complexity and operator ergonomics through cloud models with an improved multi-objective hybrid Grey Wolf Optimiser [35].

Despite recent progress, EoL-EV battery DSP still presents several challenges. Penalty-based constraint handling is easy to implement but may retain infeasible candidates until evaluation, wasting computation. Feasibility-preserving encoding, repair, and decoding reduce such candidates but require problem-specific design [8]. Fixed operator-selection rules may also respond poorly to changing search states, motivating adaptive selection [13,32].

While adaptive operator selection and reinforcement learning have been integrated with metaheuristics, including bandit-based selection in evolutionary algorithms [13,32], a hybrid BA with reinforcement learning for DSP [14], and a hybrid particle swarm optimisation with Q-learning (PSO-Q-learning) approach for power-battery disassembly [15], these studies mainly focus on improving search adaptability. Recent work has also combined Q-learning with complementary global and local search strategies for power-system scheduling, demonstrating the broader applicability of learning-guided multi-strategy optimisation [36]. The incorporation of process-level disassembly constraints into candidate solution generation remains largely unexplored. 

Although reinforcement-learning-based strategies have been investigated in optimisation, the application of contextual-bandit-based operator selection, using observed search-state features, with BA has received limited attention. BA was selected as the optimisation framework because its combination of scout-bee exploration and neighbourhood exploitation mechanisms provides a balance between global search and local refinement, making it well suited to discrete disassembly sequence optimisation with complex constraints. Furthermore, its modular structure facilitates the integration of both constraint-handling mechanisms and adaptive operator-selection strategies.

Recent advances in battery lifetime prediction and chemistry-aware machine learning also highlight opportunities to incorporate battery-condition information into future disassembly planning systems [37,38,39,40]. To address these challenges, the subsequent section details the proposed constraint-guided adaptive optimisation framework.

## 3. Methodology

### 3.1. Problem Formulation

#### 3.1.1. Modelling of Disassembly Constraints

**Figure 1:**
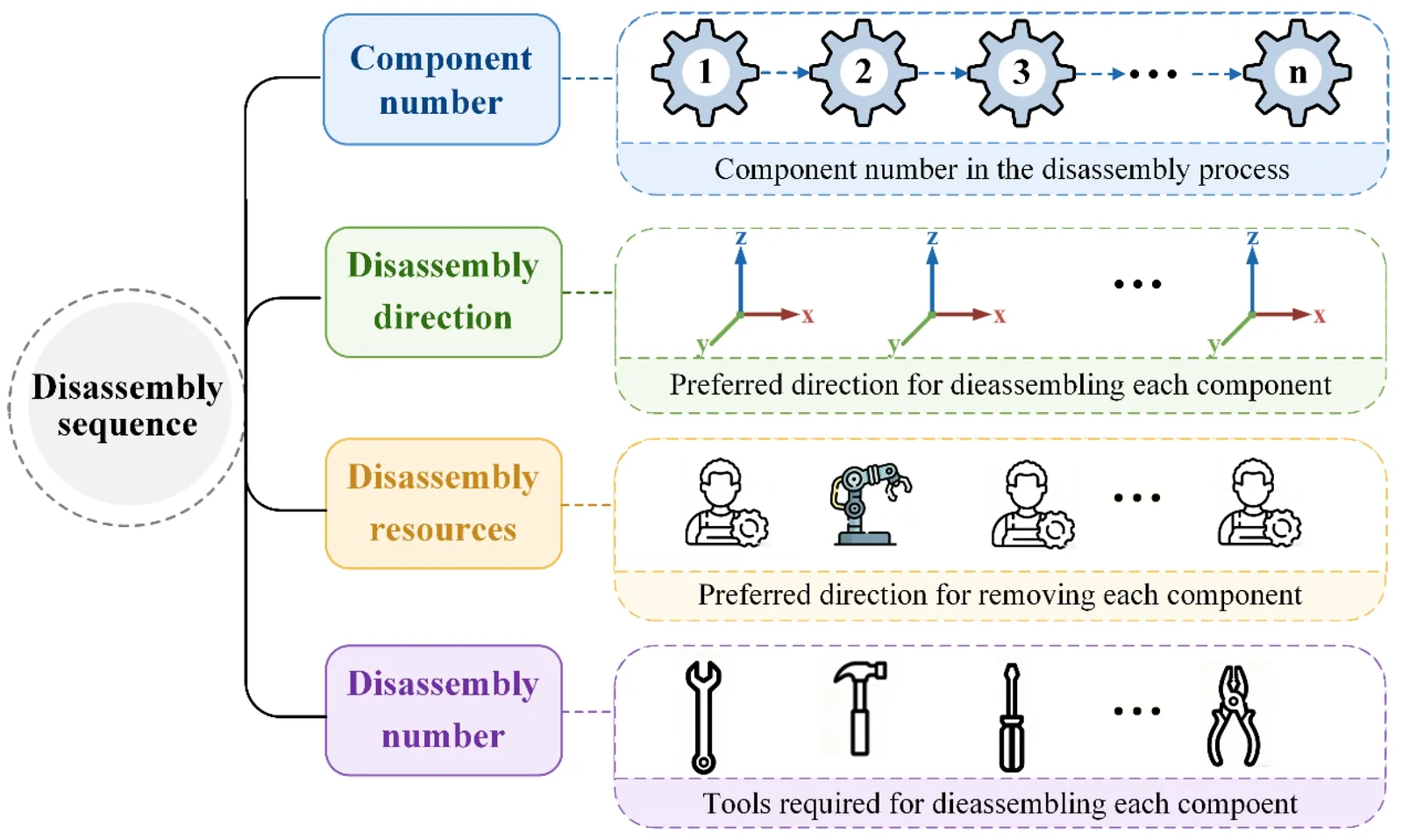
Disassembly sequence composition.

Disassembly constraints are designed to ensure the physical and logical viability of the sequences generated. A disassembly sequence is represented as a multi-dimensional ordered vector. A serial disassembly mode is adopted, in which only one agent, an operator or a robotic arm, is permitted to perform disassembly operations on a single component at a time. It is further assumed that every component can be removed completely and without damage, that is, the disassembly is non-destructive. As shown in Figure 1, each component in a human–robot collaborative disassembly sequence is described by its component number, disassembly direction, tool, and disassembly resource. Four collaborative resource modes are specified: manual disassembly (labelled 1), robotic disassembly (labelled 2), human disassembly with robotic placement (labelled 3), and robotic disassembly with human placement (labelled 4). Six fundamental disassembly directions are defined, covering the positive and negative directions along the X, Y, and Z axes.

Physical feasibility is the main constraint; no collisions are permitted during component removal. A six-directional interference matrix represents the geometric constraints between components. The components of a product to be disassembled can be denoted as:

(1) $$N=\{n_{1},n_{2,}\cdots ,n_{m}\}$$

**Figure 2:**
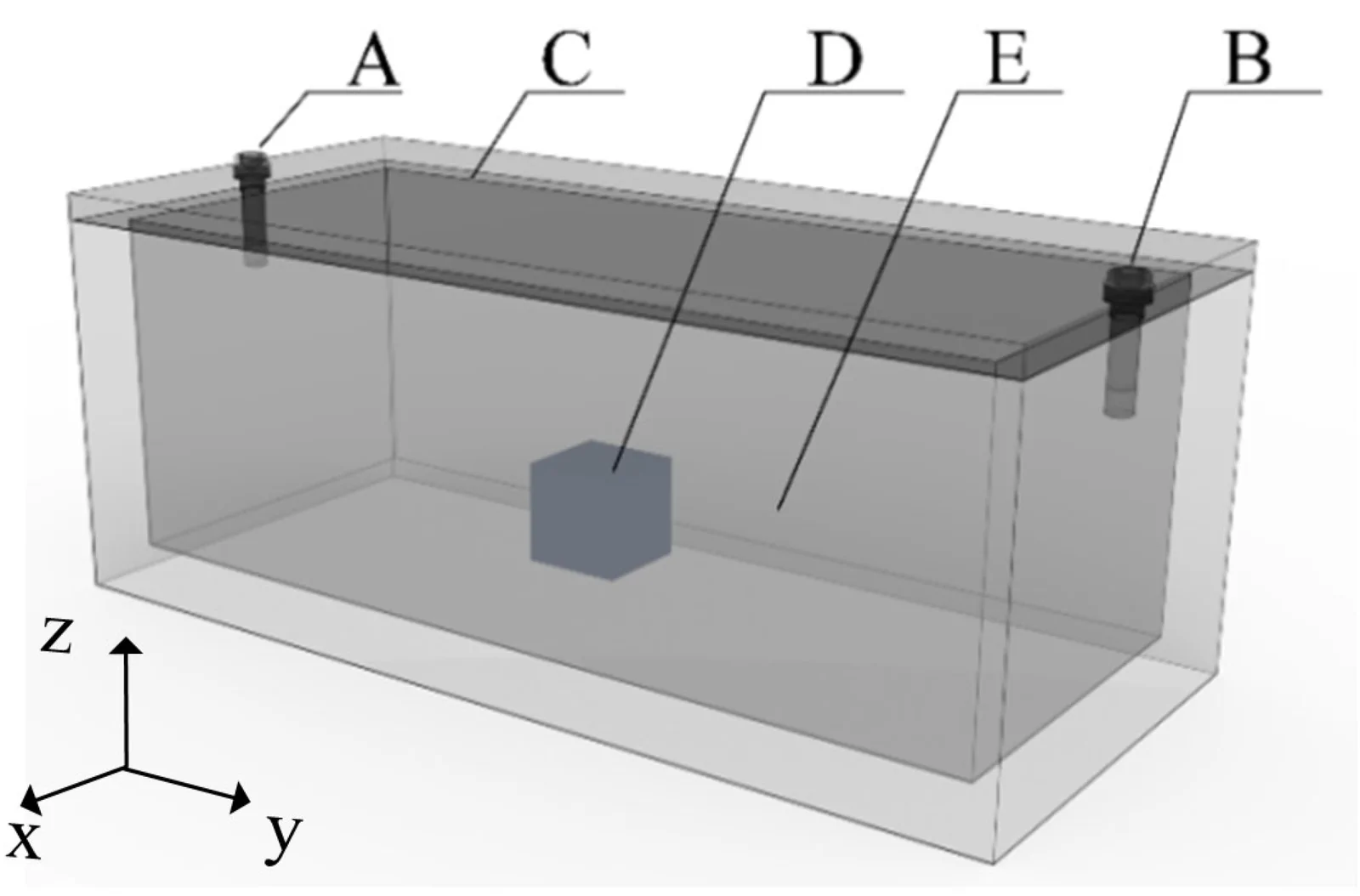
Modelling of the interference matrix.

The interference matrix of the product along the X+ direction is denoted as $S_{x+}$, which is an m×m matrix. An entry of 0 indicates that the component $n_{i}$ can be removed along the X+ direction while component $n_{j}$ exists, whereas an entry of 1 indicates that component $n_{j}$ obstructs the removal of component $n_{i}$ in that direction. As shown in Figure 2, the six disassembly interference matrices of the product can be constructed in Equation (2). Component A cannot be disassembled along the Z-direction with Component C in place.

(2) $$\begin{array}{ccc} S_{x+}=\begin{array}{cc} & \begin{array}{ccccc} A & B & C & D & E \end{array}\\ \begin{array}{c} A\\ B\\ C\\ D\\ E \end{array} & \left[\begin{array}{ccccc} 0 & 1 & 1 & 1 & 1\\ 1 & 0 & 1 & 1 & 1\\ 1 & 1 & 0 & 0 & 0\\ 1 & 1 & 0 & 0 & 1\\ 1 & 1 & 0 & 1 & 0 \end{array}\right] \end{array} & S_{x-}=\begin{array}{cc} & \begin{array}{ccccc} A & B & C & D & E \end{array}\\ \begin{array}{c} A\\ B\\ C\\ D\\ E \end{array} & \left[\begin{array}{ccccc} 0 & 1 & 1 & 1 & 1\\ 1 & 0 & 1 & 1 & 1\\ 1 & 1 & 0 & 0 & 0\\ 1 & 1 & 0 & 0 & 1\\ 1 & 1 & 0 & 1 & 0 \end{array}\right] \end{array} & S_{y+}=\begin{array}{cc} & \begin{array}{ccccc} A & B & C & D & E \end{array}\\ \begin{array}{c} A\\ B\\ C\\ D\\ E \end{array} & \left[\begin{array}{ccccc} 0 & 1 & 1 & 1 & 1\\ 1 & 0 & 1 & 1 & 1\\ 1 & 1 & 0 & 0 & 0\\ 1 & 1 & 0 & 0 & 1\\ 1 & 1 & 0 & 1 & 0 \end{array}\right] \end{array}\\ S_{y-}=\begin{array}{cc} & \begin{array}{ccccc} A & B & C & D & E \end{array}\\ \begin{array}{c} A\\ B\\ C\\ D\\ E \end{array} & \left[\begin{array}{ccccc} 0 & 1 & 1 & 1 & 1\\ 1 & 1 & 1 & 1 & 1\\ 1 & 1 & 0 & 0 & 0\\ 1 & 1 & 0 & 0 & 1\\ 1 & 1 & 0 & 1 & 0 \end{array}\right] \end{array} & S_{z+}=\begin{array}{cc} & \begin{array}{ccccc} A & B & C & D & E \end{array}\\ \begin{array}{c} A\\ B\\ C\\ D\\ E \end{array} & \left[\begin{array}{ccccc} 0 & 0 & 0 & 0 & 0\\ 0 & 0 & 0 & 0 & 0\\ 1 & 1 & 0 & 0 & 0\\ 1 & 1 & 1 & 0 & 1\\ 1 & 1 & 1 & 0 & 0 \end{array}\right] \end{array} & S_{z-}=\begin{array}{cc} & \begin{array}{ccccc} A & B & C & D & E \end{array}\\ \begin{array}{c} A\\ B\\ C\\ D\\ E \end{array} & \left[\begin{array}{ccccc} 0 & 1 & 1 & 1 & 1\\ 1 & 0 & 1 & 1 & 1\\ 1 & 1 & 0 & 1 & 1\\ 1 & 1 & 0 & 0 & 1\\ 1 & 1 & 0 & 1 & 0 \end{array}\right] \end{array} \end{array}$$

The six resulting disassembly interference matrices along the orthogonal directions are fused into the interference matrix $S_{x,y,z}$, as shown in Equation (3). The result column contains 6-bit binary strings. Each bit is obtained by a logical OR operation on the same bit of all elements in the corresponding row of the spatial interference matrix $S_{x,y,z}$. “0” means the part can be disassembled along the corresponding direction, while “1” means it cannot.

(3) Sx,y,z=  A     B    C  D EABCDE000000 111101 111101 111101 111101 111101 000000 111101 111101 111101 111111 111111 000000 000001 000001111111 111111 000010 000000 111111111111 111111 000010 111101000000Result111101111101111111111111111111

Besides physical interference, the rule base encodes additional hard constraints, each with a clear engineering justification on safety and structural grounds, as follows:

(1) High-voltage safety priority. The high-voltage harness assembly and Battery Management System (BMS) must be disassembled before any battery modules. This guarantees that the entire module disassembly process is carried out in a de-energised state, thereby significantly reducing the risk of operator electric shock and thermal runaway caused by high-voltage short circuits.

(2) Cooling system priority. Cooling system components must be removed before battery modules to prevent coolant leakage during module disassembly, which could otherwise cause high-voltage circuit insulation failure and phase-to-phase short circuits.

(3) Structural stability principle. The lower housing must be designated as the final component for disassembly. As the load-bearing foundation of the entire battery pack, premature removal of the lower housing before all internal components have been extracted would result in structural instability and potential component drop hazards. This constraint maintains overall structural integrity throughout the disassembly process.

Strict constraints are integrated into the composite interference matrix for unified representation. The resulting enhanced matrix functions are directly incorporated into subsequent feasible sequence generation.

#### 3.1.2. Optimisation Objectives

Three optimisation objectives are considered: time cost (T), payment cost (P), and utility value (U). Because time cost and payment cost are minimisation objectives, whereas utility value is a maximisation objective (with higher values indicating better ergonomic suitability), all three metrics are normalised to values between 0 and 1 using predefined min–max bounds before weighted aggregation. Specifically, forward min–max normalisation is applied, while reverse min–max normalisation is applied to convert it into a utility deficit. The aggregated objective function is defined as:

(4) $$C_{total}=\omega_{1}\bar{T}+\omega_{2}\bar{P}+\omega_{3}\bar{U}$$

where $\bar{T}$, $\bar{P}$ represent the normalised time cost and payment cost, respectively; $\bar{U}$ represents the normalised utility deficit; and $\omega_{1}$, $\omega_{2}$, and $\omega_{3}$ are the corresponding objective weights. For reinforcement learning, the weighted objective value is multiplied by a constant scaling factor solely for reward computation, without affecting the optimisation process. Unless otherwise specified, all reported objective values refer to the normalised weighted objective value before reward scaling. Although this formulation produces a single aggregated objective value rather than a Pareto-optimal solution set, the weighting mechanism allows operational preferences to be explicitly incorporated, thereby facilitating trade-offs among time cost, payment cost, and utility. To accommodate different operational needs, four disassembly modes are defined: balanced mode, economic mode, efficient mode, and ergonomic mode. The disassembly cost includes labour and robot maintenance. Disassembly utility is assessed based on the level of compatibility between tasks and human–robot resources [32].

(1) Time cost

The total time is the sum of the basic disassembly time ($bt(x_{i})$) of individual components, component placement time (pt), robot pose-changing time (dt), tool-switching time (tt), and end-effector movement time (mt), which can be calculated by:

(5) $$f(X)=\sum_{i=1}^{n-1}bt(x_{i})+\sum_{i=1}^{n-1}pt(n_{i})+\sum_{i=1}^{n-1}dt(n_{i},n_{i+1})+\sum_{i=1}^{n-1}tt(n_{i},n_{i+1})+\sum_{i=1}^{n-1}mt(n_{i},n_{i+1})$$

where dt is the time of the robot’s disassembly direction changing from component $n_{i}$ to component $n_{i+1}$. It is assumed that the direction-changing time is 0 s, 1 s, or 2 s.

(6) $$dt_{i}=\left\{\begin{array}{ll} 0, & {dir}_{n_{i}}={dir}_{n_{i+1}}\\ 1, & |{dir}_{n_{i}}-{dir}_{n_{i+1}}|=90{}^{\circ}\\ 2, & |{dir}_{n_{i}}-{dir}_{n_{i+1}}|=180{}^{\circ} \end{array}\right.$$

where $tt(n_{i},n_{j})$ is the time for the disassembly tool to switch from $n_{i}$ to $n_{j}$, and $mt(n_{i},n_{j})$ is the time for the end-effector change between two components. The time taken for the end-effector path movement is calculated by dividing the path length by the linear velocity of the end effector. The path length ($ml_{i,j}$) between two modules can be calculated via the Euclidean distance.

(7) $$ml_{i,j}=\sqrt{{\left(x_{i}-x_{j}\right)}^{2}+{\left(y_{i}-y_{j}\right)}^{2}+{\left(z_{i}-z_{j}\right)}^{2}}$$

(2) Payment cost

The total payment cost is determined by the time spent by human operators and robots on the disassembly task. Operator payment costs include salary and training expenses. The operator’s pay ($P_{h}$) (dollars per second) is defined as:

(8) $$P_{h}=\frac{P_{salary}+P_{train}}{3600N}$$

where $P_{salary}$ is the annual salary of an operator, $P_{train}$ is the cost of an operator’s one-year training, and N is the number of hours an operator works in one year. The robot cost ($P_{r}$) can be calculated by:

(9) $$P_{r}=\frac{P_{equipment}+P_{maintenance}}{3600N_{h}N_{y}}+\frac{P_{electricity}}{3600N_{h}}$$

where $P_{electricity}$ is the annual electricity consumption cost of the robot. The robot is assumed to have a service life ($N_{y}$ years), and working time ($N_{h}$ hours per year). The initial purchase cost is $P_{equipment}$ and the total maintenance cost is $P_{maintenance}$ during its operational period. The total payment cost can be calculated by:

(10) $$P_{t}=\sum_{i=1}^{m}(P_{h}T_{i}^{h}+P_{r}T_{i}^{r})$$

where $T_{i}^{h}$ and $T_{i}^{r}$ are the operational time of the human operator and the robot for disassembly tasks, respectively.

(3) Utility value

The utility of human operators is assessed using multi-dimensional ergonomic indicators. Muscle fatigue is modelled to forecast physical exertion decline during extended work, with postural comfort, force magnitude, and duration evaluated thoroughly. Task difficulty is determined by tool requirements, reachability, and positioning accuracy, and a repetition factor is included to consider fatigue build-up from long-term repetitive tasks. The utility value (Uh) of human operators can be calculated by:

(11) $$U_{h}=0.4f_{H,erg}+0.3f_{H,dif}+0.2f_{H,rep}+0.1f_{H,mus}$$

where $f_{H,erg}$ is the ergonomic fitness value, which can be obtained by:

(12) $$f_{H,erg}=0.4\times f_{basic}+0.3\times f_{material}+0.1\times (f_{twist}+f_{lateral}+f_{reach})$$

where $f_{basic}$ is the basic posture fitness, and $f_{material}$ is the material handling fitness. $f_{twist}$, $f_{lateral}$, and $f_{reach}$ are the total fitness values for torso twist, lateral bending, and reach range, respectively. The disassembly difficulty fitness ($f_{H,dif}$) can be calculated as:

(13) $$f_{H,dif}=\frac{f_{requirement}+f_{access,d}+f_{access,l}+f_{position}}{4}$$

where $f_{requirement}$ denotes the tool requirement fitness, $f_{access,d}$ is the dimensional accessibility fitness of joints or slots, $f_{access,l}$ denotes the positional accessibility fitness value, and $f_{position}$ is the tool positioning accuracy fitness value. $f_{H,rep}$ is the operation repetition adaptability coefficient, quantifying the impact of the repetition frequency of the same disassembly task on operator efficiency and fatigue status. Values close to 1 indicate minimal fatigue build-up and high adaptability, while lower values suggest greater performance decline.

$f_{H,mus}$ is the muscle fatigue fitness value. Its calculation relies on a dynamic muscle fatigue model and is determined by the ratio of Muscle Endurance Time (MET) to Maximum Endurance Time (MAT) under a specific load.

(14) $$f_{H,mus}=\left\{\begin{array}{cc} \frac{MAT}{MET}, & MAT<MET\\ 0, & MAT>MET \end{array}\right.$$

where MET is the duration for muscle force to decay to the level required by the task load, and MAT is the maximum sustainable operation time that muscles can maintain under a specific load. The robot utility value is assessed based on capability matching, which can be calculated by:

(15) $$U_{r}=0.33\times f_{R,dif}+0.33\times f_{R,reach}+0.33\times f_{R,pay}$$

where $f_{R,dif}$ is the disassembly difficulty value. It is assigned based on the required motion dimension of the robotic end effector: 1 for unidirectional motion, 0.8 for bidirectional motion, and 0.6 for multidirectional motion. $f_{R,reach}$ is the accessibility fitness value, which is determined based on the ratio between the component spatial coordinate and the maximum working range of the robot. The coefficient reaches its maximum at a ratio of 0.6 and decreases as the ratio deviates from 0.6, thereby preventing task failure due to components exceeding their working range. $f_{R,pay}$ is the load capacity fitness coefficient, which reflects the degree of matching between the robot’s load capacity and the component weight ($W_{p}$).

(16) $$f_{R,reach}=\left\{\begin{array}{cc} \begin{array}{c} 0,\\ 1-\frac{R_{p}-W_{p}}{R_{p}},\\ 0.5, \end{array} & \begin{array}{c} R_{p}<W_{p}\\ 0.5<\frac{W_{p}}{R_{p}}<1\\ R_{p}>W_{p} \end{array} \end{array}\right.$$

Finally, for an end-of-life battery pack comprising N components, the overall utility value (U) associated with a complete collaborative disassembly sequence is formulated as the arithmetic mean of the capability-matching and ergonomic adaptability scores across all disassembly tasks:

(17) $$U=\frac{1}{N}\sum_{i=1}^{N}U_{\mathrm{task}}(i),\;\;\;\;\mathrm{where}\;U_{\mathrm{task}}(i)=\left\{\begin{array}{ll} U_{h}(i), & \mathrm{if}\;\mathrm{component}\;i\;\mathrm{is}\;\mathrm{assigned}\;\mathrm{to}\;\mathrm{human}\\ U_{r}(i), & \mathrm{if}\;\mathrm{component}\;i\;\mathrm{is}\;\mathrm{assigned}\;\mathrm{to}\;\mathrm{robot} \end{array}\right.\;$$

By maximising the overall utility value U (or, equivalently, minimising the normalised utility deficit $\bar{U}$ in Equation (4)), the optimisation agent strategically assigns repetitive or physically demanding operations to the robot while preserving operator ergonomics.

### 3.2. Feasible Sequence Generation

#### 3.2.1. Disassembly Priority Classification

**Figure 3:**
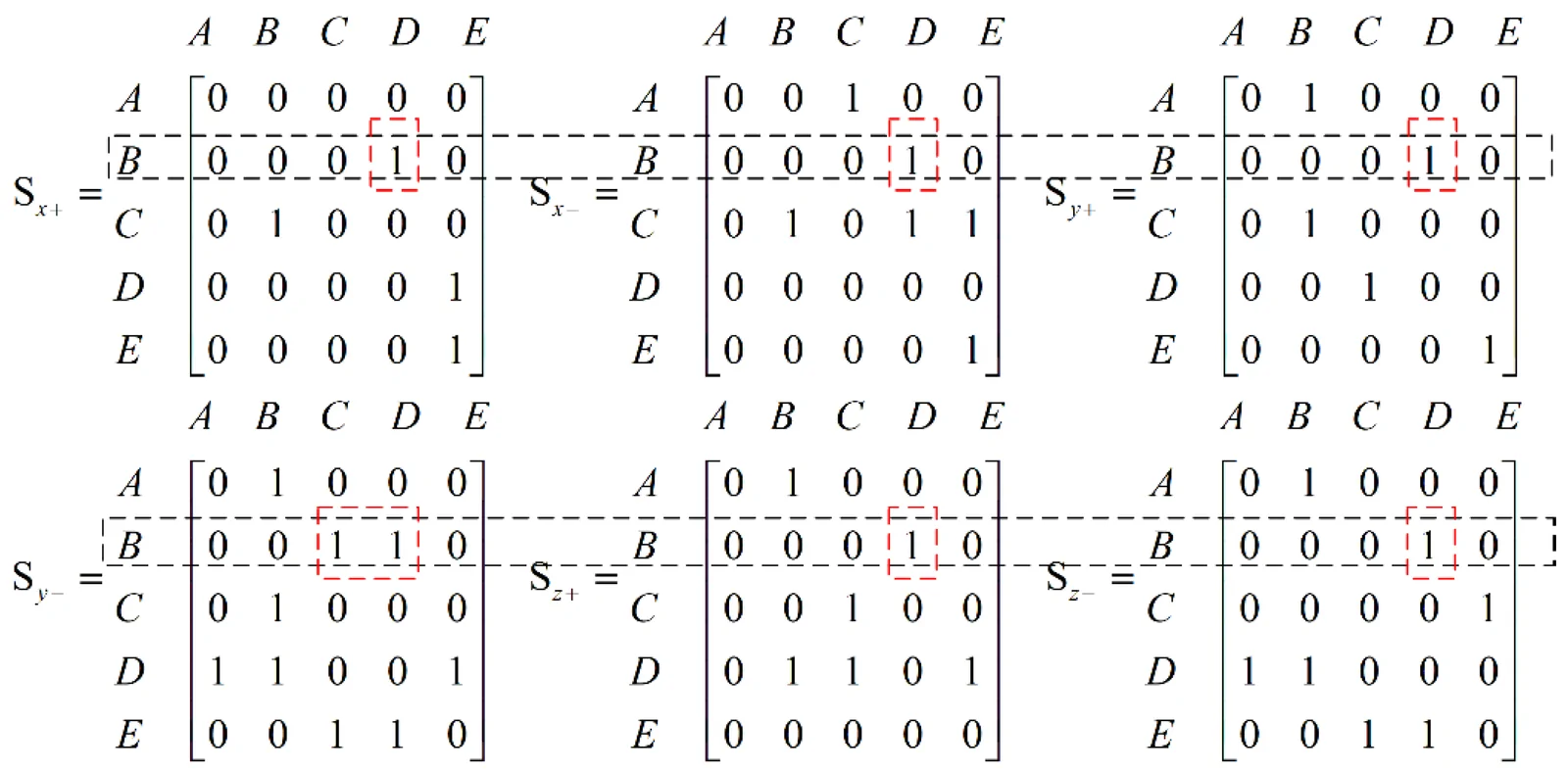
Degree of interference.

To quantitatively assess the disassembly priority of components, a multi-factor weighted calculation method is developed based on composite interference matrices and component attributes. The interference degree, disassembly difficulty, and hazard coefficient are comprehensively evaluated. After normalisation, the disassembly priority is determined through fixed-weight summation, as shown in Equation (18). A higher value indicates a greater disassembly priority.

(18) $$priority(i)=0.4\cdot S_{b}(i)+0.25\cdot S_{d}(i)+0.35\cdot S_{t}(i)$$

where $S_{b}(i)$ is the degree of interference imposed on component i, which can be obtained by:

(19) $$S_{b}(i)=\frac{1}{N_{b}(i)+1}$$

where $N_{b}(i)$ is the total number of unique components obstructing the component, which is obtained by counting and deduplicating the obstruction relationships across the six spatial interference matrices. As illustrated in Figure 3, the number of unique obstructing components for Component B is 2. The dashed boxes indicate the interference relationships being analysed in each step.

The disassembly difficulty coefficient ($S_{d}(i)$) can be calculated using Equation (20). Dif(i) denotes the dimensionless hazard level of component i. The hazard levels of different component types are defined in Table 1. Components with lower hazard risks are assigned higher priority values to ensure safe sequential disassembly.

(20) $$S_{d}=1-Dif(i)$$

Furthermore, to eliminate dimensional discrepancies and scale differences across components, the disassembly time priority coefficient ($S_{t}(i)$) is mapped onto a uniform dimensionless interval between 0 and 1 using standard min–max normalisation, as defined in Equation (21). $T_{d}(i)$ is the disassembly time of the component i. $T_{\max}$ and $T_{\min}$ are the maximum and minimum disassembly times of all components, respectively.

(21) $$S_{t}(i)=\frac{T_{\max}-T_{d}(i)}{T_{\max}-T_{\min}}$$

#### 3.2.2. Feasible Initial Sequence Generation

After quantifying component disassembly priorities, an iterative process is used to generate feasible initial disassembly sequences. The sequence generation depends on a composite spatial interference matrix integrated with hard constraints. A mechanism that integrates a greedy strategy with probability perturbation is used to construct sequences.

In each iteration, all remaining components are traversed to identify candidates. Only those with valid free disassembly directions are chosen. To prevent the loss of sequence diversity due to the dominance of high-priority components, a smooth, weighted probability model is employed. A minimum base selection probability is assigned to each candidate. Priority values are converted into selection weights, so components with higher priorities have greater chances of being selected. Nonetheless, all candidates retain some possibility of being chosen. This method balances the algorithm’s exploration and exploitation capabilities. Regarding direction selection, engineering standards for top-down battery pack disassembly are strictly followed. The positive Z-direction is preferred and selected only when available. If not, a direction is randomly chosen from the remaining free options, ensuring alignment with real production practices. This preference is treated as a fixed process constraint rather than an optimisable parameter. Once a component is chosen, the interference matrix is dynamically updated, removing interference constraints between it and other components. The component is then marked as disassembled to prevent reselection. Subsequently, blocker counts, free directions, and priorities for the remaining components are recalculated. Components that were blocked are unlocked for the next candidate selection. This process repeats until all components are included in the sequence.

**Figure 4:**
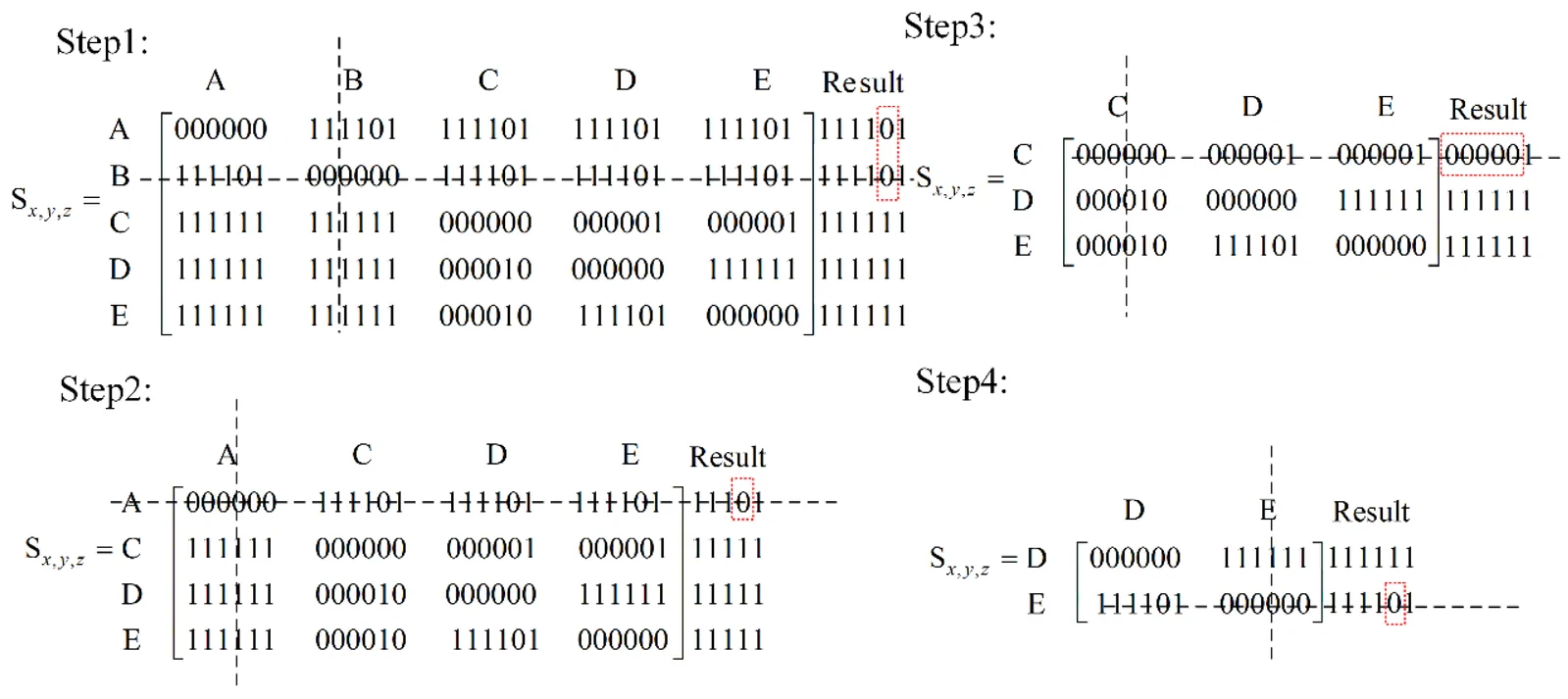
Process of feasible sequence generation.

The iterative process of sequence generation is illustrated by the dynamic updating of the composite interference matrix ($S_{x,y,z}$) for five components, as shown in Figure 4. In the figure, the dashed red boxes indicate the candidate components eligible for removal at each step, whereas the black dashed lines indicate the components selected for disassembly. In Step 1, Components A and B are removable as indicated by 0 values in their result column, and Component B is selected first. After removing B and updating the matrix in Step 2, Component A becomes the only removable part and is disassembled next. In Step 3, with A and B removed, Component C is identified as removable and selected accordingly. Finally, in Step 4, the matrix is updated once more, and Components D and E are disassembled in sequence until all parts have been removed.

Building on the generated disassembly sequence, human–robot collaborative resource scheduling and time quantification are further conducted. Executors are assigned to each component using a probabilistic strategy, where manual operation is given a higher baseline probability to leverage its superior adaptability to on-site uncertainties. This initial configuration remains adjustable and will be refined during subsequent optimisation. Tool allocation is achieved by indexing predefined arrays by part number, automatically matching each component to the specific tools required by the operator or robotic arm. For time estimation, a Euclidean distance matrix is computed for all component pairs based on their 3D coordinates. Travel times are calculated using preset velocity standards of 25 mm/s for the robotic arm and 30 mm/s for the human operator. Movement durations between consecutive tasks are accumulated segmentally, with travel time attributed to the relevant executor only when the task is not performed solely by that agent. The final output is a feasible initial solution encompassing the complete disassembly sequence, direction assignments, executor allocation, and time costs.

### 3.3. LinUCB-BA for Sequence Optimisation

**Figure 5:**
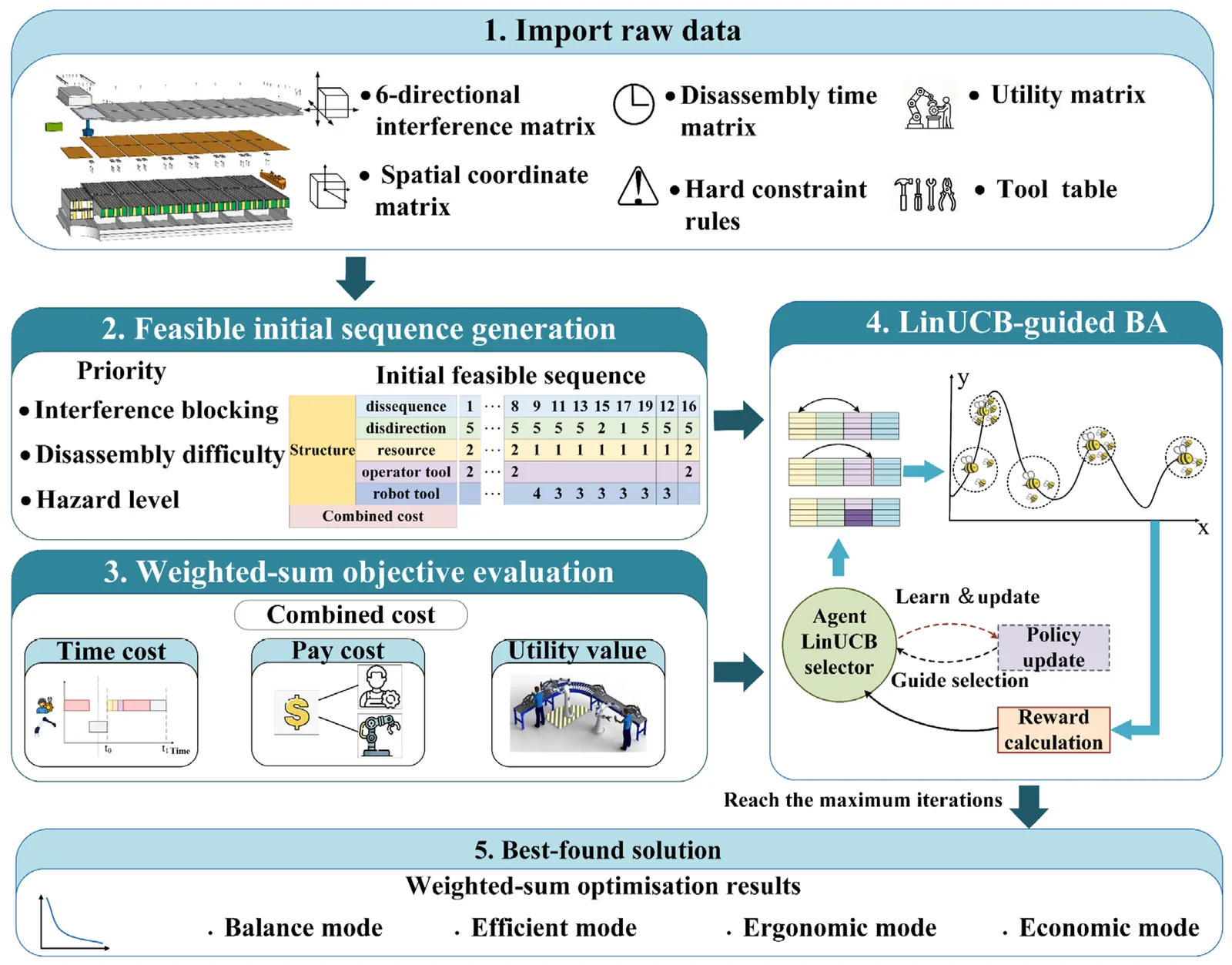
Workflow of the proposed LinUCB-guided BA framework for disassembly sequence optimisation.

Figure 5 illustrates the overall workflow of the proposed disassembly sequence optimisation method, which integrates LinUCB with the BA. The method comprises five sequential modules: raw data import, feasible initial sequence generation, weighted-sum objective evaluation, LinUCB-enhanced BA optimisation, and output of the mode-specific best-found sequence.

Like the original BA, the proposed LinUCB-BA adopts the collective foraging behaviour of honeybee colonies as a population-based optimisation framework that balances local exploitation and global exploration. This mechanism is well suited to EoL-EV battery disassembly sequence planning, where promising solutions must be refined while maintaining exploration of new feasible regions under complex engineering constraints.

In this framework, each candidate disassembly sequence is regarded as a food source, while its weighted objective value represents the corresponding nectar quality. Local neighbourhood searches around elite and selected sites are performed by recruited bees. The waggle dance mechanism is represented by LinUCB-based adaptive operator selection, where the swap, insert, and mutation operators are selected according to observed search-state features and historical rewards. Scout bees conduct global exploration, replacing uncompetitive solutions with newly generated feasible sequences, thereby maintaining population diversity. Through this integration, the system adaptively balances intensification and diversification during constrained disassembly sequence optimisation.

#### 3.3.1. Algorithm Process

The improved BA in this study includes three main phases: initialisation, neighbourhood search, and global search. The detailed implementation process is outlined as follows (Algorithm 1).
**Algorithm 1.** LinUCB-Enhanced Bees Algorithm (LinUCB-BA)**Input: Constraint data; iter, mt, ne, nb, nre, nrb, α0, γ** **Output:** Global best scheme *Best_global* 1: Initialise LinUCB state model; 2:  Set exploration rate *α* ← *α0*; 3:  Generate *mt* initial bees to form population *P*; 4:  Evaluate fitness f(*P*); 5:  Sort *P* in ascending order; 6:  *Best_global* ← *P1*; 7:  **for** *it* = 1 to *iter* **do** 8:     Extract environment state *S_t*; 9:     // Phase 1 & 2: Elite and Selected Sites Exploration 10:     **for**
*i* = 1 to *ne + nb* **do** 11:           **if**
*i* ≤ *ne* **then** 12:             *nBees* ← *nre*; 13:         **else** 14:             *nBees* ← *nrb*; 15:         **end if** 16:         **for** *j* = 1 to *nBees* **do** 17:             Select operator m via LinUCB(*S_t*, *α*); 18:             *x′* ← PerformBeeDance(*P_i*, *m*); 19:             Evaluate fitness f(*x′*); 20:             Compute reward *R* from f(*x′*); 21:             Update LinUCB parameters using *S_t* and reward *R*; 22:             **if** f(*x′*) < f(*P_i*) **then** 23:                 *P_i* ← *x′*; 24:             **end if** 25:         **end for** 26:     **end for** 27:     // Phase 3: Global Scouting and Population Update 28:     Sort *P* according to fitness; 29:     **for** *i* = *ne + nb + 1 to mt* do 30:     Reinitialise *P_i* randomly; 31:     **end for** 32:     Sort *P* again; 33:     if f(*P1*) < f(*Best_global*) then 34:         *Best_global* ← *P1*; 35:     **end if** 36: **end for** 37: **return**
*Best_global*;

(1) Initialisation

The composite interference matrices fused with the hard-constraint rule base are loaded, and the disassembly priority score of each component is calculated comprehensively based on interference constraints, disassembly difficulty, and disassembly time. The key parameters of the algorithm are defined in Table 2.

The core parameters for site selection and bee colony allocation are defined based on the population size to maintain a consistent search structure. The total number of scout bees in the foraging population (mt) is set to half of the initial scout bee population size ($n_{s}$). The number of elite sites ($n_{e}$) is set to $n_{s}$/5, and the remaining selected sites are defined as non-elite best sites ($n_{b}=m_{t}-n_{e}$). For the employed bee allocation rule for sites, six employed bees are allocated to each elite site ($n_{re}=6$), and three employed bees are allocated to each non-elite best site ($n_{rb}=3$). These proportional settings were used to maintain the same balance between local exploitation and global exploration across different population sizes. Elite sites were allocated twice as many employed bees as non-elite sites to intensify the search around promising solutions, while the reinitialisation of the lowest-ranked half of the population retained global exploration. These ratios were fixed rather than independently optimised in the present study.

The LinUCB contextual model is initialised with an exploration coefficient of 2.0. The bee structure array is pre-allocated, and the global statistical variables are also initialised. Feasible initial disassembly sequences are generated using a priority-weighted strategy based on the improved interference matrix. For each component, the free disassembly directions and the number of unique blocking components are calculated simultaneously. The scout bee population is initialised with the high-quality feasible sequences mentioned earlier, along with the assignment of disassembly directions, the allocation of human–robot resources, and tool matching for each sequence. The weighted objective value of each initial solution is then calculated.

(2) Neighbourhood search and global exploration

All scout bees are sorted based on their weighted objective value. Elite sites are chosen from this sorted group. For each elite site, neighbourhood search is carried out iteratively. Operators are selected adaptively using LinUCB. Swap, insert, and mutation operators are used to generate neighbouring solutions. New solutions are created by applying these operators. The feasibility of each solution is tested against interference constraints. The weighted objective values of the new solutions are recalculated. Global statistics are updated accordingly. LinUCB parameters are adjusted based on the rewards received. If an improvement occurs, current bees are replaced by the best new bees.

For non-elite sites, neighbourhood search is carried out similarly. Operators are selected by LinUCB without any probability constraints. New solutions are generated. Weighted objective values are calculated. Statistics are updated accordingly. Current bees are replaced by the best new bees if an improvement occurs. All bees are ranked by their weighted objective value. The lowest 50% of bees are reinitialised. New feasible sequences are created. Diversity is maintained. Local optima are avoided. The capacity for global exploration is enhanced.

#### 3.3.2. Disassembly Operators

**Figure 6:**
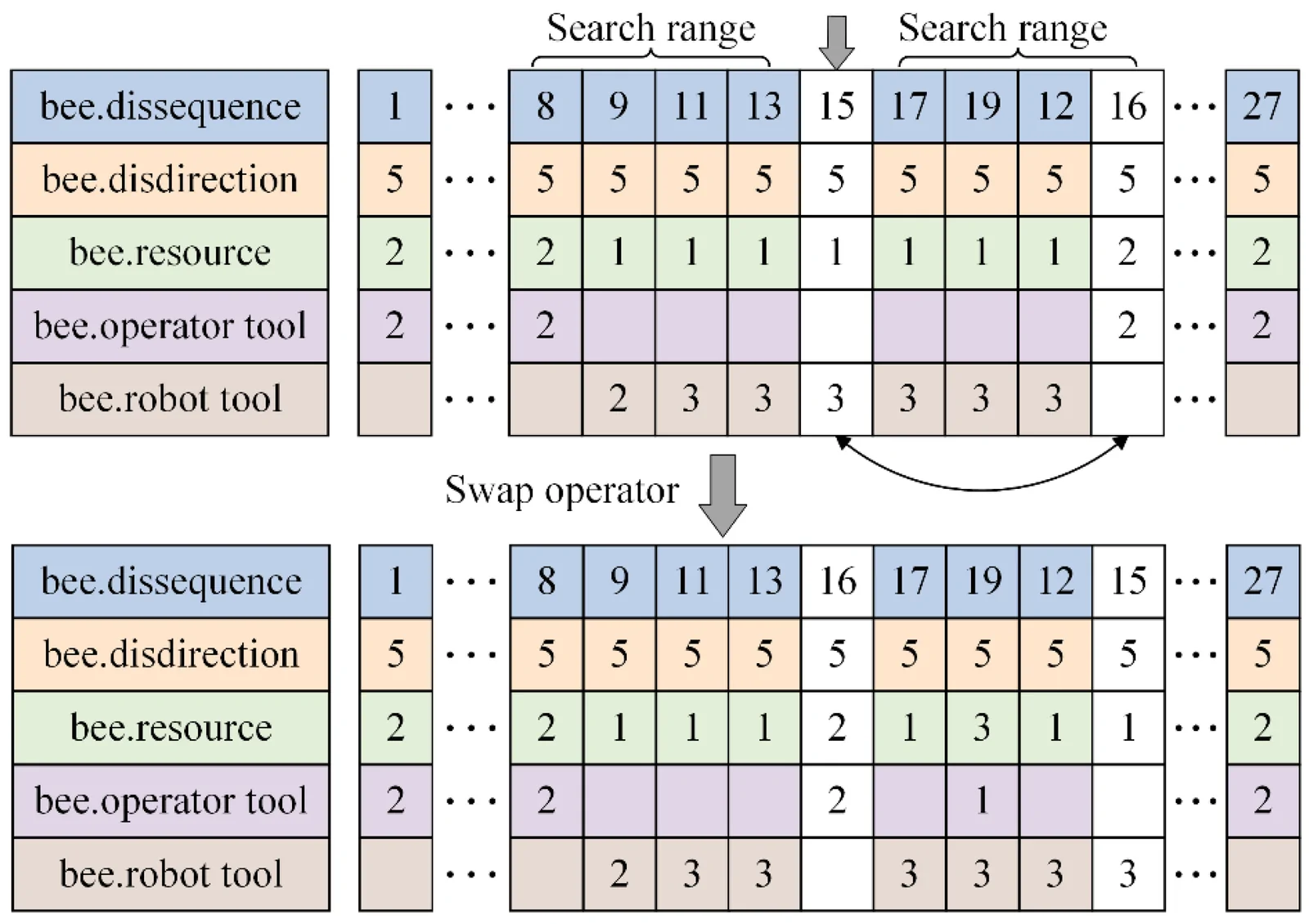
Swap operator in a disassembly solution.

A key step in the neighbourhood search phase is to modify the disassembly sequence by selecting different operators. Considering that disassembly sequence optimisation involves both component ordering and resource assignment, three complementary operators—swap, insert, and mutation—are adopted. Swap and insert modify the component order through local exchange and sequence relocation, respectively, whereas mutation changes the assigned disassembly resource and tool, thereby enhancing solution diversity. The swap operator randomly selects two components by generating two integers and then exchanges their disassembly order, which affects the disassembly direction and the tools used. Additionally, changes to the disassembly order will impact the robot’s movement time between components. The details of the swap operator are illustrated in Figure 6.

**Figure 7:**
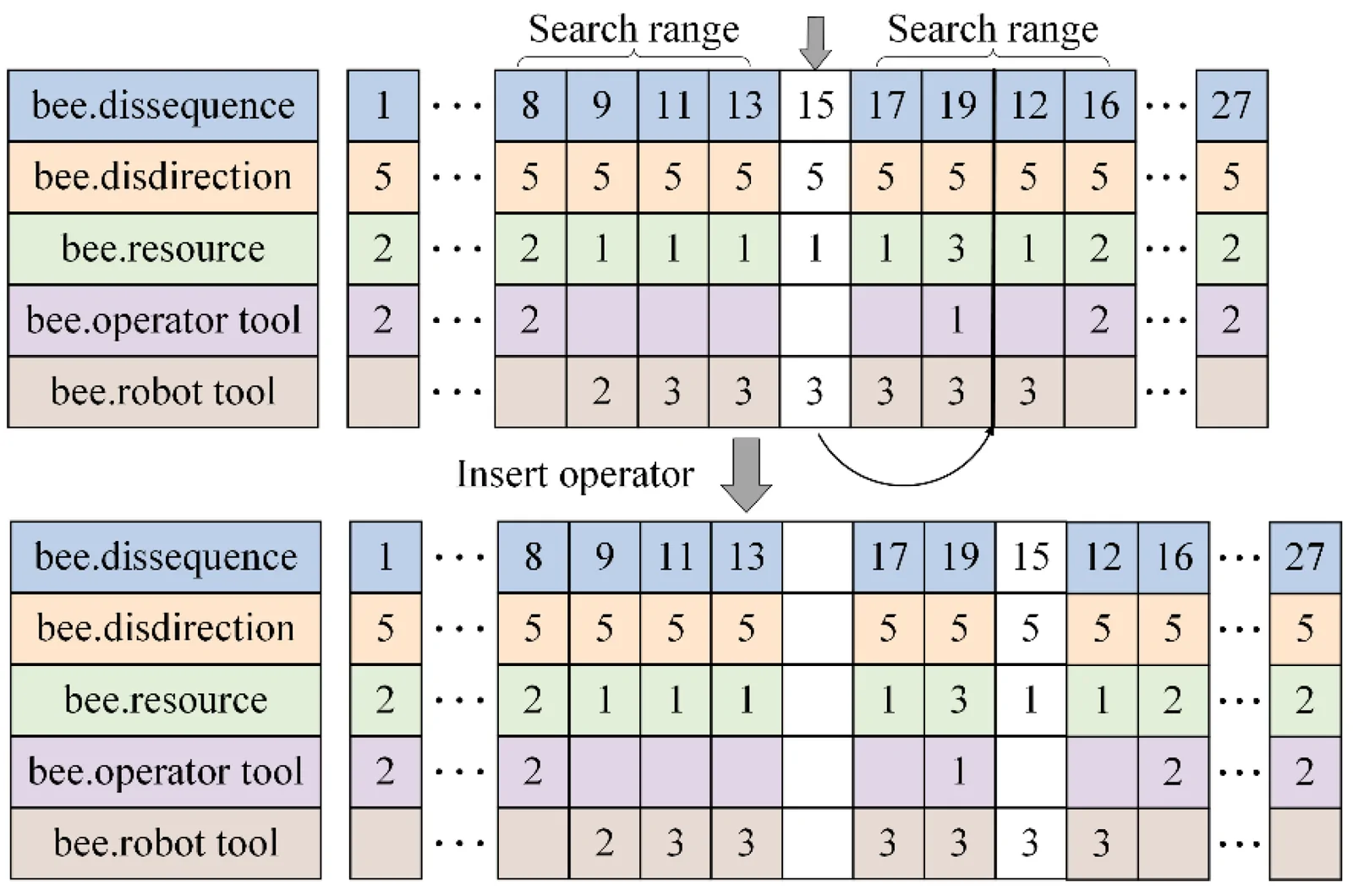
Insert operator in a disassembly solution.

**Figure 8:**
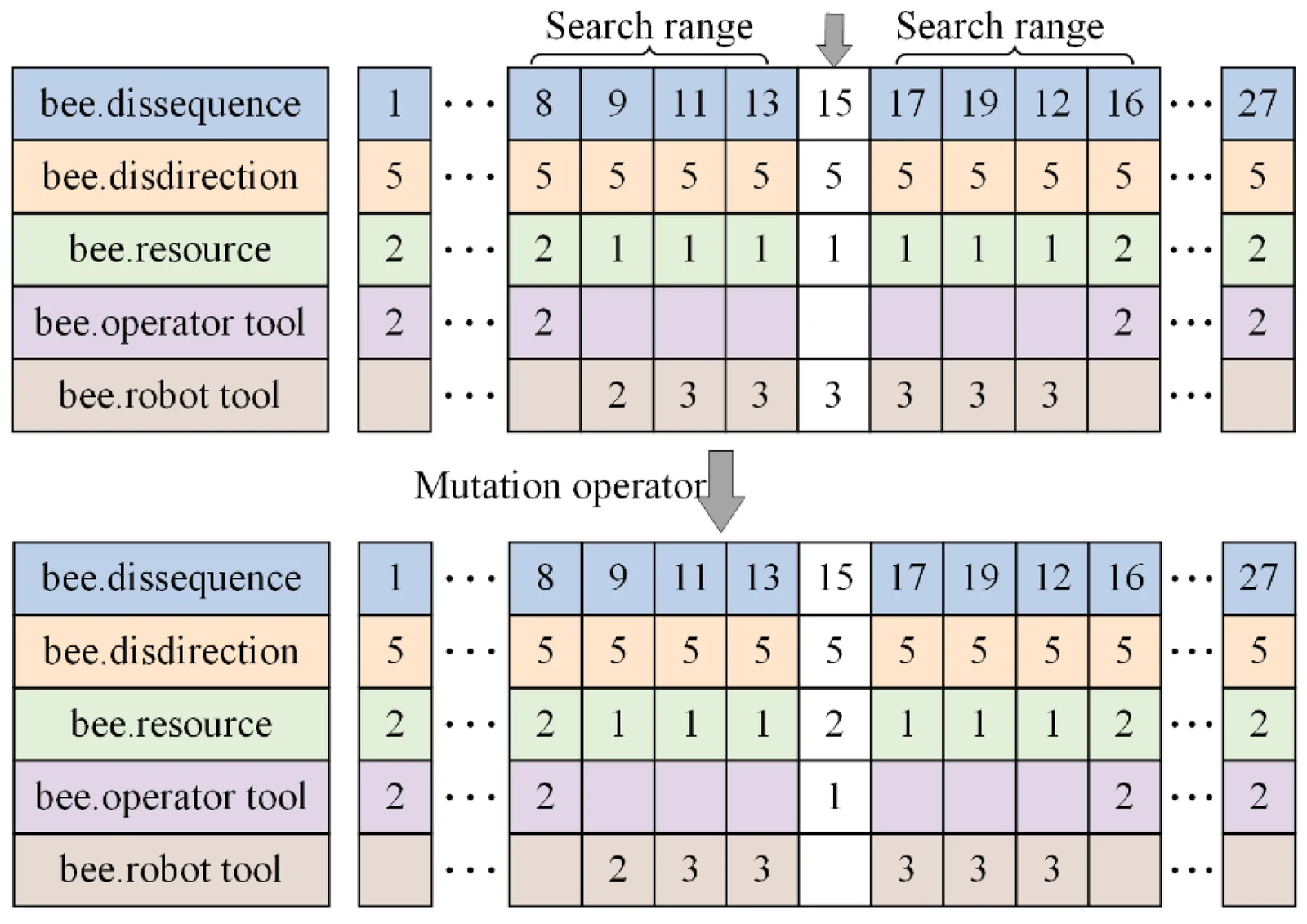
Mutation operator in a disassembly solution.

The insert operator randomly selects a component and moves it to a new position in the sequence, altering the original disassembly order and causing adaptive adjustments in the disassembly sequences of other modules. The insert operator is illustrated in Figure 7. The mutation operator randomly chooses a component, and its disassembly resource is reassigned stochastically. Consequently, the corresponding disassembly tool in the sequence is updated as shown in Figure 8. The feasibility of the newly generated disassembly sequences and directions should be verified after the neighbourhood search. If a generated sequence is infeasible, the operator will be reapplied until a feasible solution is found.

Other discrete neighbourhood operators, such as inversion, block relocation, scramble, and precedence-preserving operators, could also be incorporated into the framework. This study does not establish that the selected three-operator pool is optimal or sufficient; systematically comparing different operator-pool compositions requires a separate ablation study.

#### 3.3.3. Context-Aware LinUCB for Adaptive Operator Selection

In this study, LinUCB is introduced as a lightweight reinforcement-learning-based mechanism for adaptive operator selection rather than direct sequence generation. As operator effectiveness varies across different search states, LinUCB learns the relationship between search-state features and operator rewards, enabling dynamic operator selection through a balance between exploration and exploitation. Compared with deep reinforcement-learning methods, LinUCB requires fewer training samples and incurs lower computational overhead, making it well suited to the discrete optimisation problem considered in this study.

During the iterative optimisation process of the BA, different operators show significant performance variations across various search phases. Traditional static operator-selection strategies often cause the algorithm to become stuck in local optima or converge slowly. To address this, this section introduces an improved context-aware LinUCB algorithm for adaptive operator selection. At iteration t, to evaluate each operator (swap, insert, or mutation) i∈{1,2,3}, a 5-dimensional contextual feature vector $x_{i,t}\in R^{5}$ is constructed to quantify both the current optimisation state and the operator’s historical efficacy:

(22) $$x_{i,t}={\left[1,\omega_{t},\tau_{t},f_{i,t},g_{i,t}\right]}^{T}$$

where 1 is the intercept term, $\omega_{t}\in [0,1]$ is the normalised objective value of the current solution; $\tau_{t}=t/T_{\max}\in [0,1]$ denotes the global optimisation progress; $f_{i,t}\in [0,1]$ represents the historical feasibility success rate of operator i; and $g_{i,t}$ is the historical average percentage improvement rate achieved by operator i. Note that the intercept term establishes a baseline reward value for each operator. By preventing the underlying linear regression model from being constrained through the origin, it allows the algorithm to account for an operator’s inherent, state-independent effectiveness, thereby ensuring unbiased reward predictions even when all dynamic state features are zero.

The upper confidence bound (UCB) score $S_{i,t}$ is then calculated for each candidate operator. To ensure numerical stability against matrix singularity, an $L_{2}$ regularisation term with coefficient $\lambda =10^{-4}$ is introduced into the covariance inversion:

(23) $$S_{i,t}=\theta_{i,t}^{T}x_{i,t}+\alpha \sqrt{x_{i,t}^{T}{\left(A_{i,t}+\lambda I\right)}^{-1}x_{i,t}}$$

where $\theta_{i,t}$ is the weight vector for the operator (i). $A_{i,t}$ is the covariance matrix associated with the operator (i). The exploration coefficient is set to 2.0 and kept constant throughout the search process, providing a consistent level of exploration without manual scheduling.

After the selected operator is executed, an immediate heuristic reward ($r_{t}$) is calculated to update the LinUCB parameters. Instead of a static reward, $r_{t}$ is dynamically designed based on the physical feasibility and the relative objective improvement rate:

(24) $$r_{t}=\left\{\begin{array}{cc} \frac{\cos t_{old}-\cos t_{new}}{\cos t_{old}}\times 100\%, & \mathrm{if}\;cost_{new}<cost_{old}\\ -0.1, & \mathrm{if}\;\mathrm{feasible}\;\mathrm{but}\;\mathrm{no}\;\mathrm{improvement}\\ -1.0, & \mathrm{if}\;\mathrm{infeasible}\;(\mathrm{deadlock}) \end{array}\right.$$

Specifically, this heuristic reward ensures that operators producing infeasible solutions are heavily penalised, while those delivering significant cost savings are prioritised. To reduce the influence of outdated observations, a forgetting factor (γ=0.95) is introduced during the parameter-update phase:

(25) $$\begin{array}{l} A_{i,t}\leftarrow \gamma A_{i,t-1}+x_{i,t}x_{i,t}^{T}+(1-\gamma )\lambda_{leak}\\ b_{i,t}\leftarrow \gamma b_{i,t-1}+r_{t}x_{i,t} \end{array}$$

where $\lambda_{leak}=10^{-3}$ is a minor stability term to prevent diagonal decay collapse.

Additionally, to address the highly constrained nature of the spatial interference matrix, a forced exploration strategy (cold start) is employed during the initial 50 iterations. During this warm-up period, operators are selected with equal probability to establish an unbiased experience pool, preventing the agent from prematurely penalising high-risk operators and falling into early topological deadlocks. Following this phase, LinUCB-guided selection is activated for all sites. After the warm-up phase, an ε-greedy exploration strategy is retained. *ε* is set to 0.15 to avoid premature convergence caused by excessive exploitation.

Unless otherwise specified, α, γ, ε, population size, and other algorithm parameters remain fixed throughout all comparative experiments to ensure fair algorithm comparison.

## 4. Case Studies

To validate the effectiveness and practicality of the proposed disassembly sequence optimisation method using the LinUCB-BA, two EoL-EV battery packs with different component sizes are selected for validation experiments. The two cases represent different levels of structural complexity: the larger battery pack provides a challenging scenario with more components and complex interference relationships, while the smaller battery pack is used to evaluate the adaptability of the method under a relatively compact structure. MATLAB R2023b was used for computational experiments on a computer equipped with 16 GB of 3200 MHz DDR4 RAM and an NVIDIA GeForce RTX 3060 GPU. The experiments are divided into two main parts: initial sequence analysis and algorithm comparison.

The geometric information and spatial interference matrices of the battery packs were extracted from 3D CAD models reconstructed using publicly available battery-pack information and technical reference materials. Disassembly times, cost parameters, and ergonomic coefficients were obtained from prior studies and supplemented by reasonable engineering assumptions for simulation-based evaluation. Safety-related hard constraints were formulated according to established principles for high-voltage battery handling. No physical dismantling experiments were conducted as part of this study. Therefore, the numerical parameters used should be regarded as inputs informed by the literature and by engineering assumptions for simulation-based planning and evaluation, rather than experimentally calibrated measurements.

### 4.1. Experimental Subjects

#### 4.1.1. Case Study A

**Figure 9:**
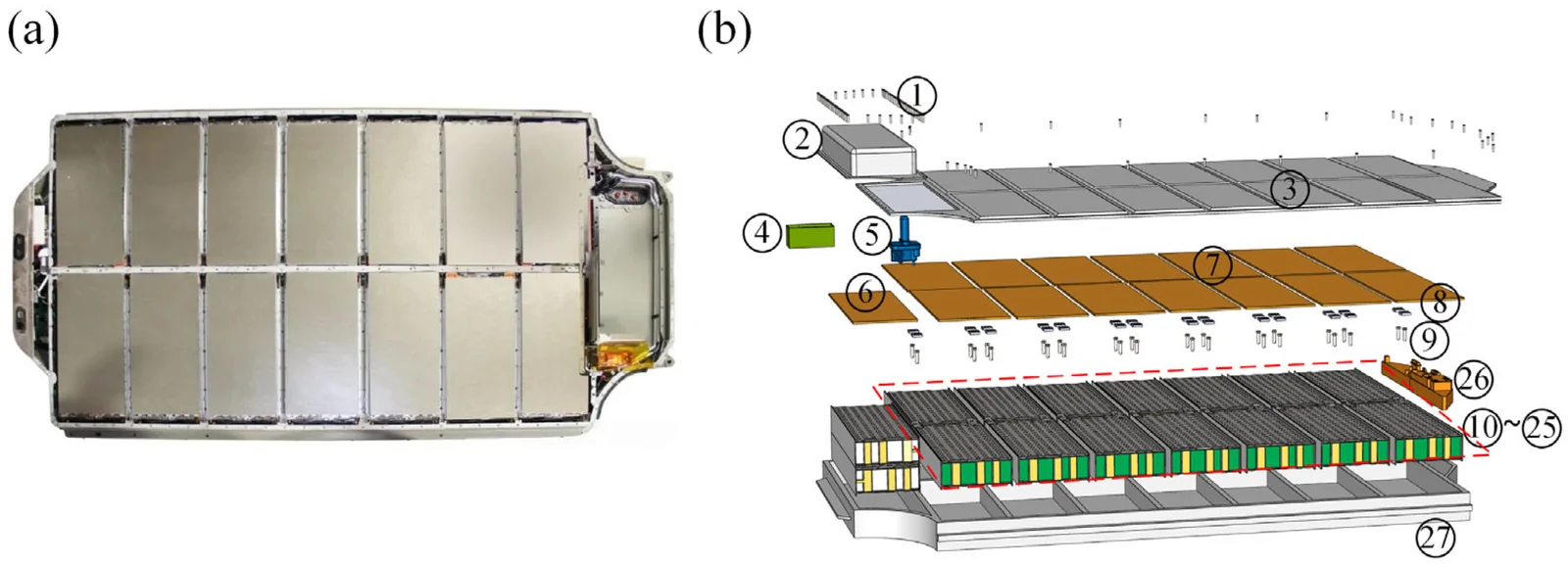
A Tesla battery pack. (**a**) Photograph. (**b**) Exploded view.

A case study of a Tesla EV battery pack was conducted to validate the proposed disassembly sequence optimisation method. Photographs and a 3D exploded view of the modules are shown in Figure 9. The labels in the figure correspond to the numbers listed in Table 3. The disassembly information for the components, including component coordinates and disassembly time taken by the robot and the operator, is listed in Table 3. Methods 1–4 correspond to four disassembly strategies: Method 1 corresponds to robot-only disassembly, Method 2 to human-only disassembly, Method 3 to one human–robot collaborative mode, and Method 4 to an alternative configuration characterised by a different task allocation strategy. These four methods were designed to evaluate the influence of resource allocation and configuration modes on overall disassembly performance. Table 4 lists the objective weight coefficients for the four disassembly optimisation modes.

#### 4.1.2. Case Study B

**Figure 10:**
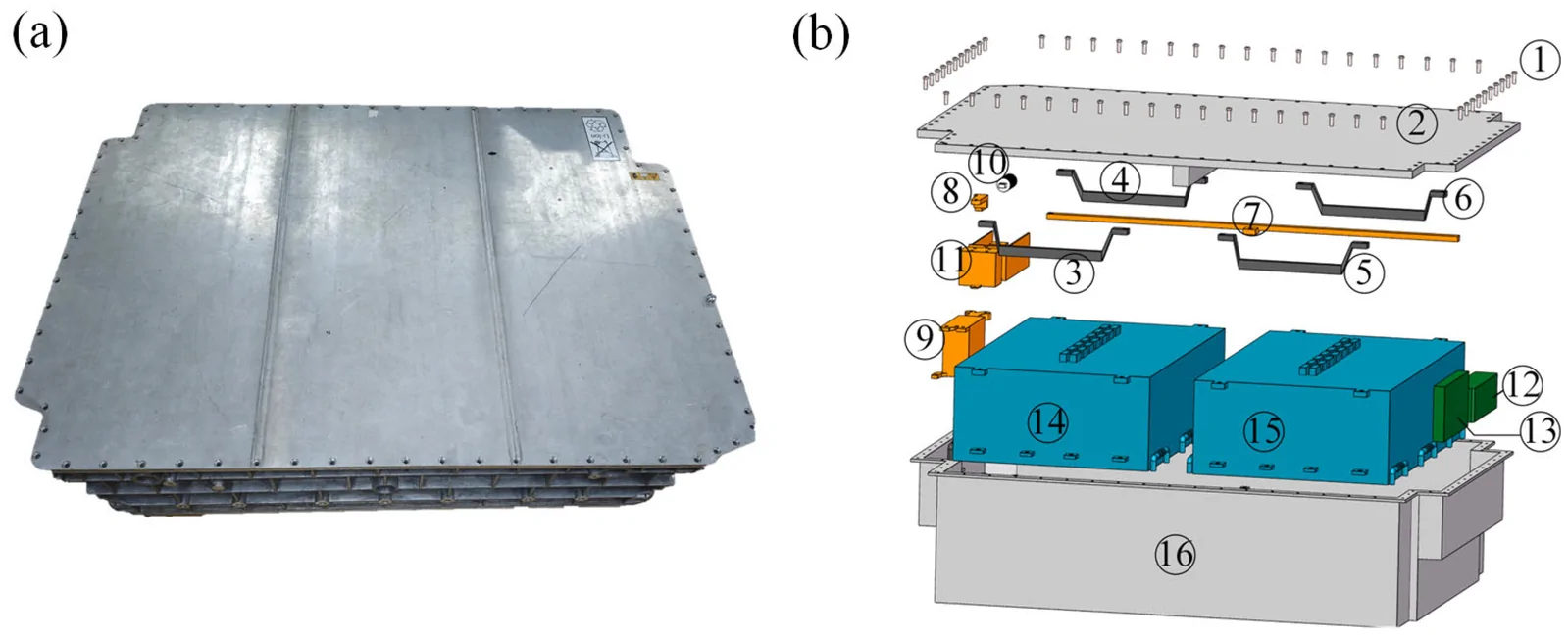
A Mercedes-Benz battery pack. (**a**) Photograph. (**b**) Exploded view.

A Mercedes-Benz battery pack mainly consisting of 16 components was used as the disassembly object. The photographs and internal structure are shown in Figure 10. The component information is listed in Table 5. The disassembly time for infeasible operations is set to 10,000 s to provide a numerical penalty.

### 4.2. Disassembly Sequence Planning Results

**Figure 11:**
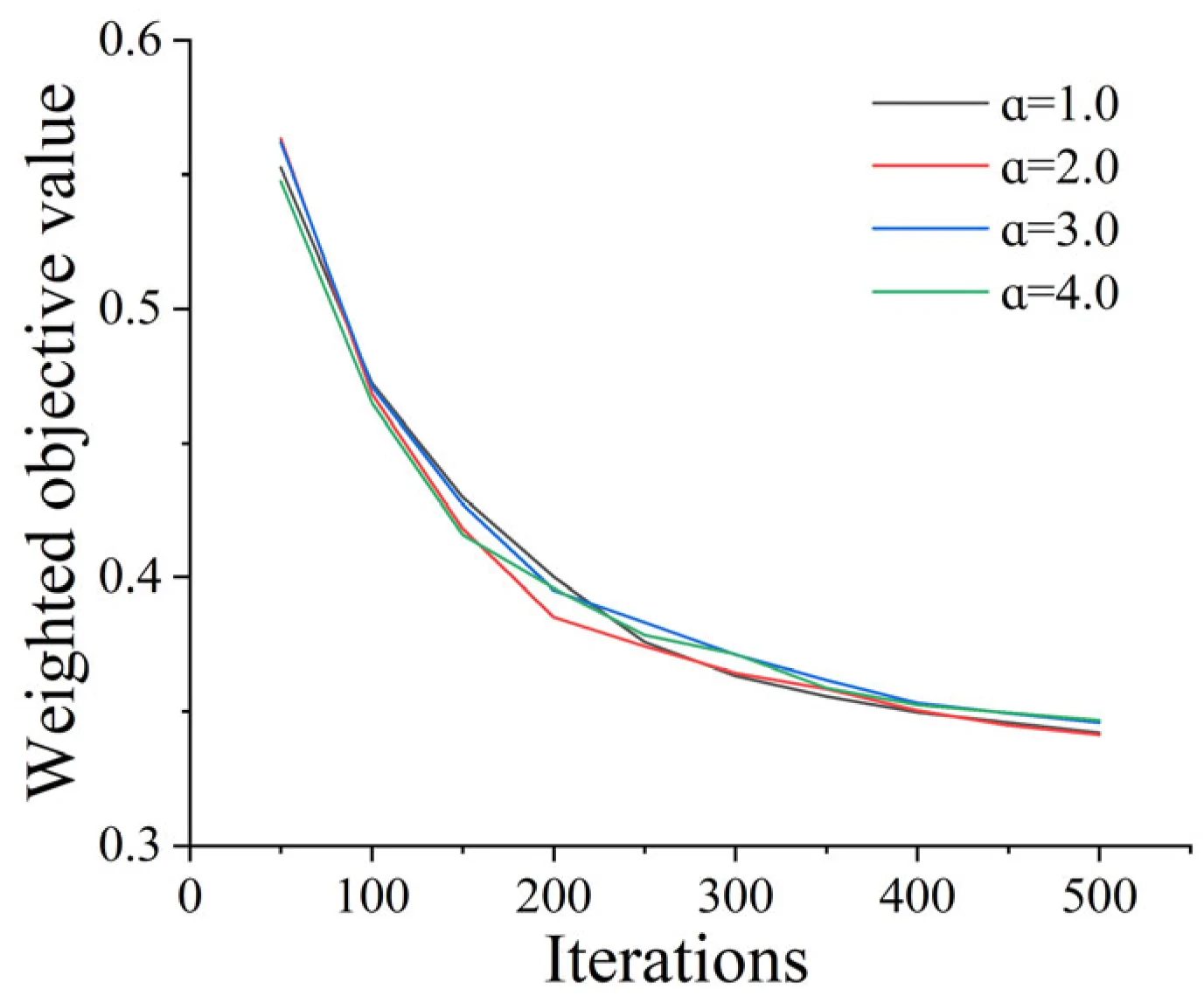
Parameter-sensitivity analysis of the exploration coefficient α.

Before evaluating the overall performance, a sensitivity analysis was conducted on Case A under the balanced mode with a population size of 50 to determine the optimal exploration coefficient $\alpha \in \left\{1.0,2.0,3.0,4.0\right\}$. For simplicity, the aggregated objective function is referred to as the weighted objective value in the following sections. As shown in Figure 11, the convergence curves across all tested values descend steadily and reach comparable objective levels by iteration 500 (ranging from 0.3413 to 0.3466), indicating that the algorithm is robust and relatively insensitive to variations in this hyperparameter. Among the tested values,   α=2.0 achieves the lowest final weighted objective value of 0.3413 at the 500th iteration, followed closely by   α=1.0 (0.3420). Consequently,   α=2.0 is adopted as the default exploration setting for all subsequent experiments.

With the baseline parameters established, to investigate the key factors influencing the algorithm’s optimisation performance, an analysis is further conducted from three perspectives: maximum iteration count, population size, and disassembly mode.

#### 4.2.1. Case Study A: Disassembly Optimisation for Tesla Battery Pack

To evaluate the optimisation performance of the proposed algorithm on the Tesla battery pack disassembly problem, Case Study A was conducted. Under the balanced disassembly mode, the effects of population size and maximum iteration number were investigated. The population size was varied from 20 to 80 with an interval of 10, while the maximum iteration number was varied from 50 to 450 with an interval of 50. For each parameter setting, 50 independent runs were performed.

**Figure 12:**
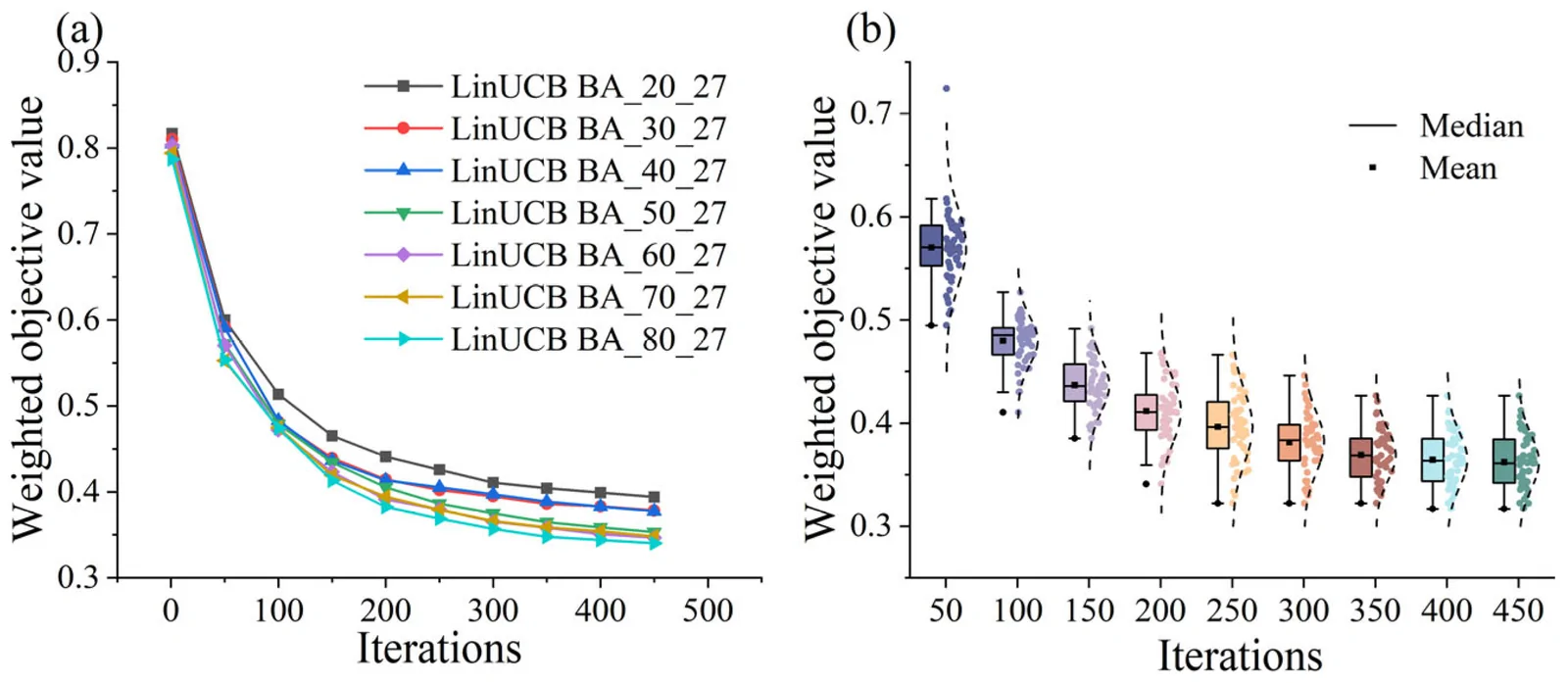
Parameter-sensitivity analysis of the proposed LinUCB-BA in Case Study A. (**a**) Mean weighted objective values under different population sizes. (**b**) Distribution of weighted objective values under different iteration numbers.

As shown in Figure 12a, the mean weighted objective value decreases as the population size increases. Considering solution quality and consistency across the subsequent experiments, a population size of 80 was selected as the unified setting for the remaining analyses. Figure 12b shows that the weighted objective value gradually decreases with increasing iteration number and becomes relatively stable after approximately 400 iterations, indicating that the algorithm has reached a stable optimisation state. Therefore, a maximum of 450 iterations was adopted for the subsequent within-method analyses of LinUCB-BA, unless otherwise specified.

The engineering performance metrics obtained at the selected iteration settings (population size = 80 and maximum iteration number = 450) are summarised in Table 6. The average disassembly time, operational cost, and human–robot utility are 1951.73 s, $688.26, and 85.70%, respectively. The corresponding standard deviations are 34.39 s, $19.65, and 0.32%, indicating that the proposed algorithm provides consistent engineering performance over repeated trials.

Given the NP-hard nature of the disassembly sequence planning problem and the complexity arising from multi-criteria optimisation objectives, spatial interference relationships, and safety constraints, obtaining mathematically guaranteed global optima for large-scale instances remains computationally challenging. Therefore, the solutions reported in this study are regarded as the best-found feasible solutions identified by the proposed heuristic algorithm within the predefined search budget rather than exact global optima.

**Figure 13:**
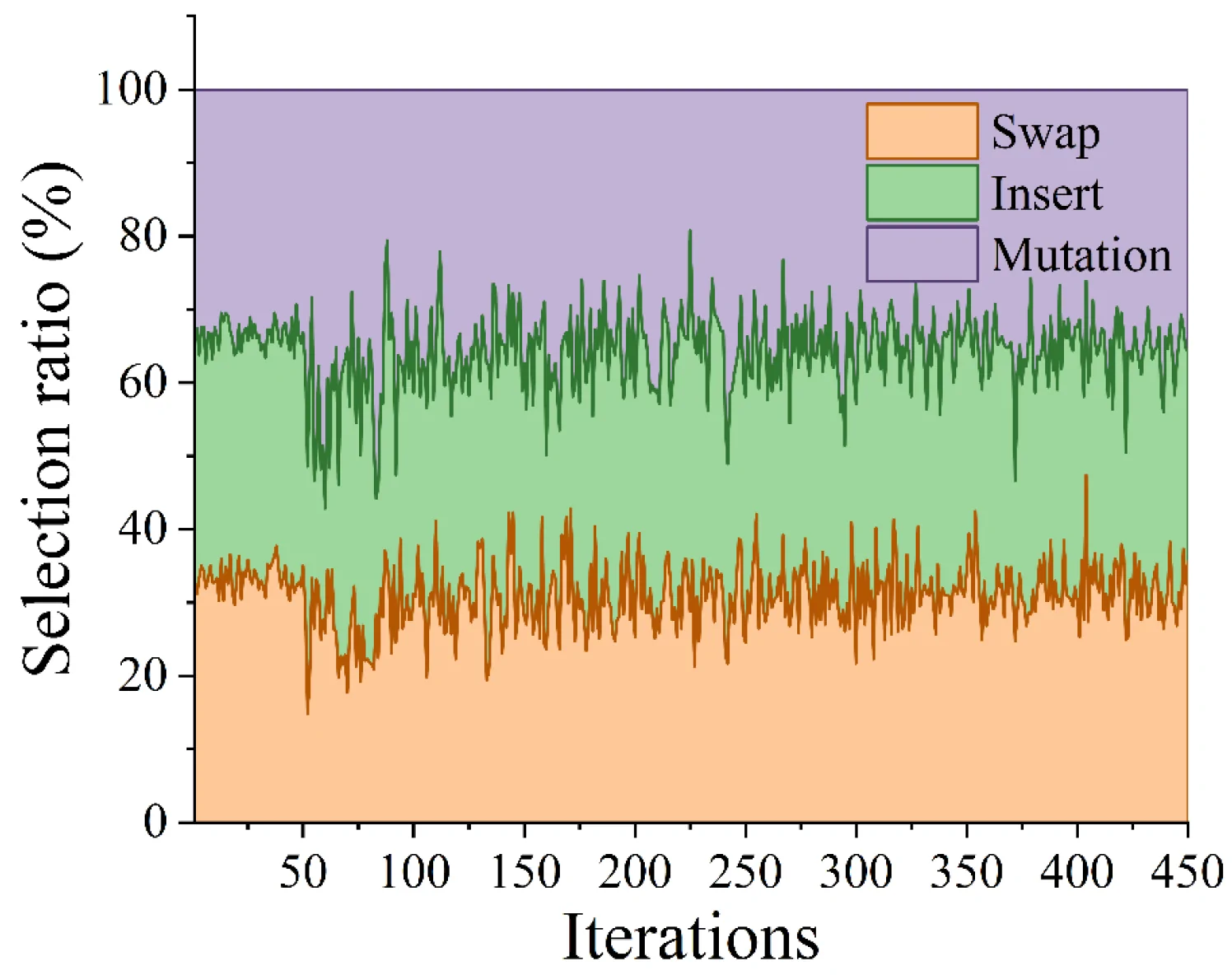
Dynamic evolution of selection ratios for three operators in Case Study A.

This stable optimisation behaviour can be attributed to the adaptive operator-selection mechanism. To illustrate its internal dynamics, Figure 13 presents the evolution of the selection ratios of the swap, insert, and mutation operators during the iterative process. Following the 50-iteration warm-up phase established in the methodology, the selection ratios dynamically adjust according to the observed feasibility and improvement performance of different operators. The subsequent changes in operator preferences demonstrate the capability of the proposed mechanism to balance exploration and exploitation during the optimisation process.

Keeping the population size at 80, the four disassembly modes were then compared, with the best-found solution for each mode reported in Table 7.

#### 4.2.2. Case Study B: Disassembly Optimisation for Mercedes-Benz Battery Pack

Case Study B was conducted using the same parameter settings as Case Study A to further validate the applicability of the proposed algorithm to battery packs with different structural characteristics. Compared with Case Study A, the Mercedes-Benz battery pack contains fewer components, resulting in a relatively smaller search space.

**Figure 14:**
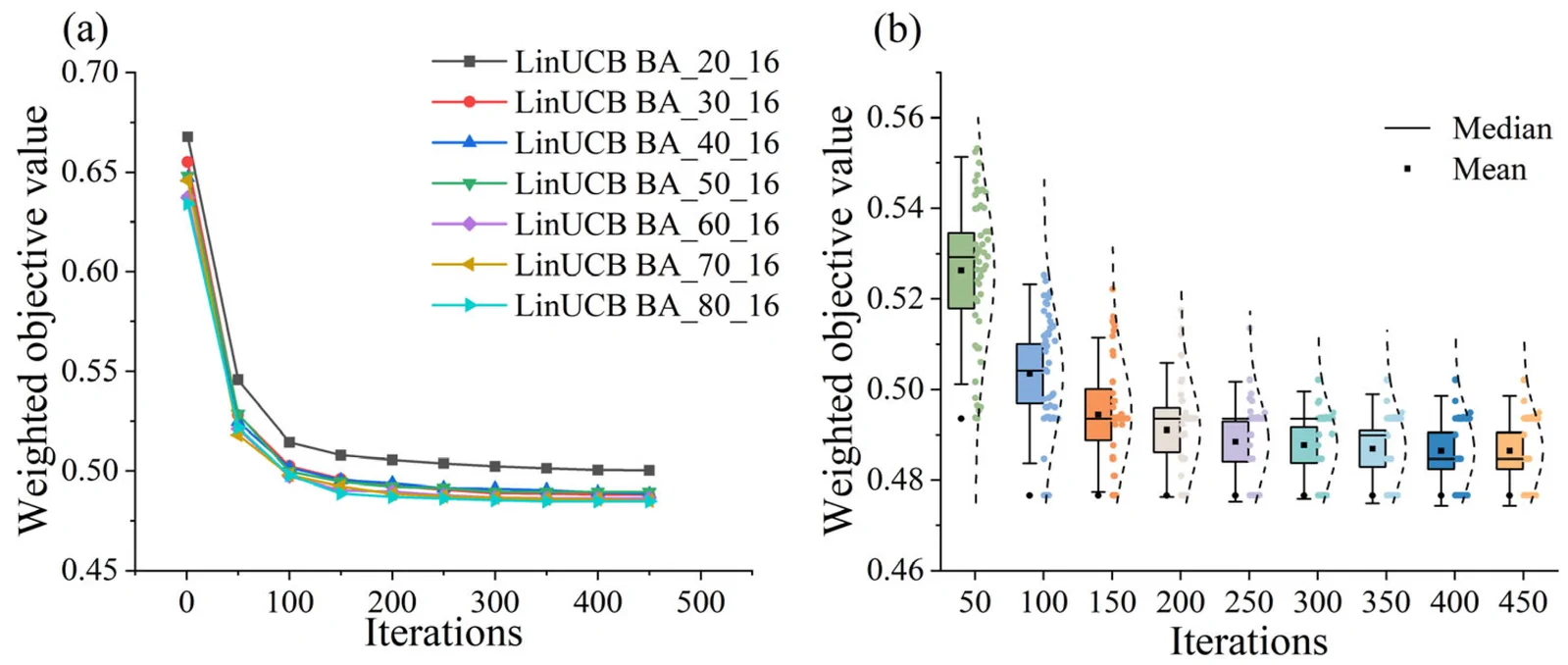
Parameter-sensitivity analysis of the proposed LinUCB-BA in Case Study B. (**a**) Mean weighted objective values under different population sizes. (**b**) Distribution of weighted objective values under different iteration numbers.

As shown in Figure 14a, increasing the population size improves solution quality, but the additional improvement becomes modest as the population grows. Considering consistency with Case Study A and the unified setting used in the subsequent experiments, a population size of 80 was retained. Figure 14b shows that the weighted objective value decreases with increasing iteration number and becomes relatively stable after approximately 200–300 iterations, demonstrating the robustness of the proposed algorithm. Therefore, the maximum iteration number was set to 450 for the subsequent experiments.

The corresponding engineering performance metrics are summarised in Table 8. The average disassembly time, operational cost, and human–robot utility are 728.79 s, $393.02, and 79.85%, respectively. The standard deviations are 4.60 s, $11.19, and 0.24%, indicating consistent practical performance of the obtained disassembly sequences across repeated runs. Furthermore, the average convergence generation is 200.6 ± 84.8, and the average computational time per run is 56.91 ± 4.48 s.

**Figure 15:**
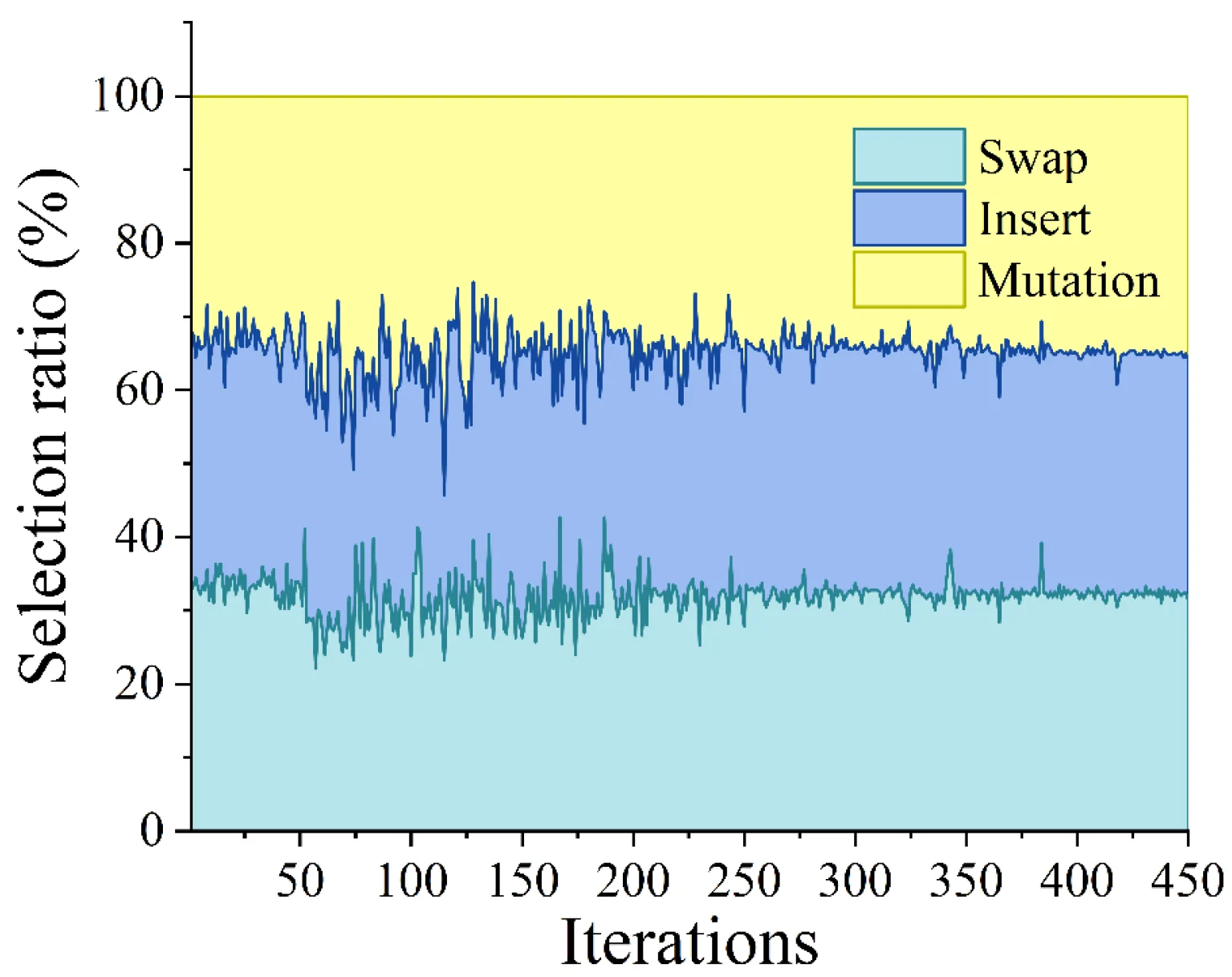
Dynamic evolution of selection ratios for three operators in Case Study B.

In Case B, Figure 15 illustrates the adaptation of operator selection during optimisation. After an initial equal-probability phase, LinUCB dynamically adjusts operator preferences according to operator performance, resulting in a more stable selection pattern in later iterations.

The best-found solutions for Case Study B across the four disassembly modes are outlined in Table 9, demonstrating the framework’s adaptability to varying operational preferences.

### 4.3. Algorithm Comparison Experiment

#### 4.3.1. Initial Sequence Analysis

**Figure 16:**
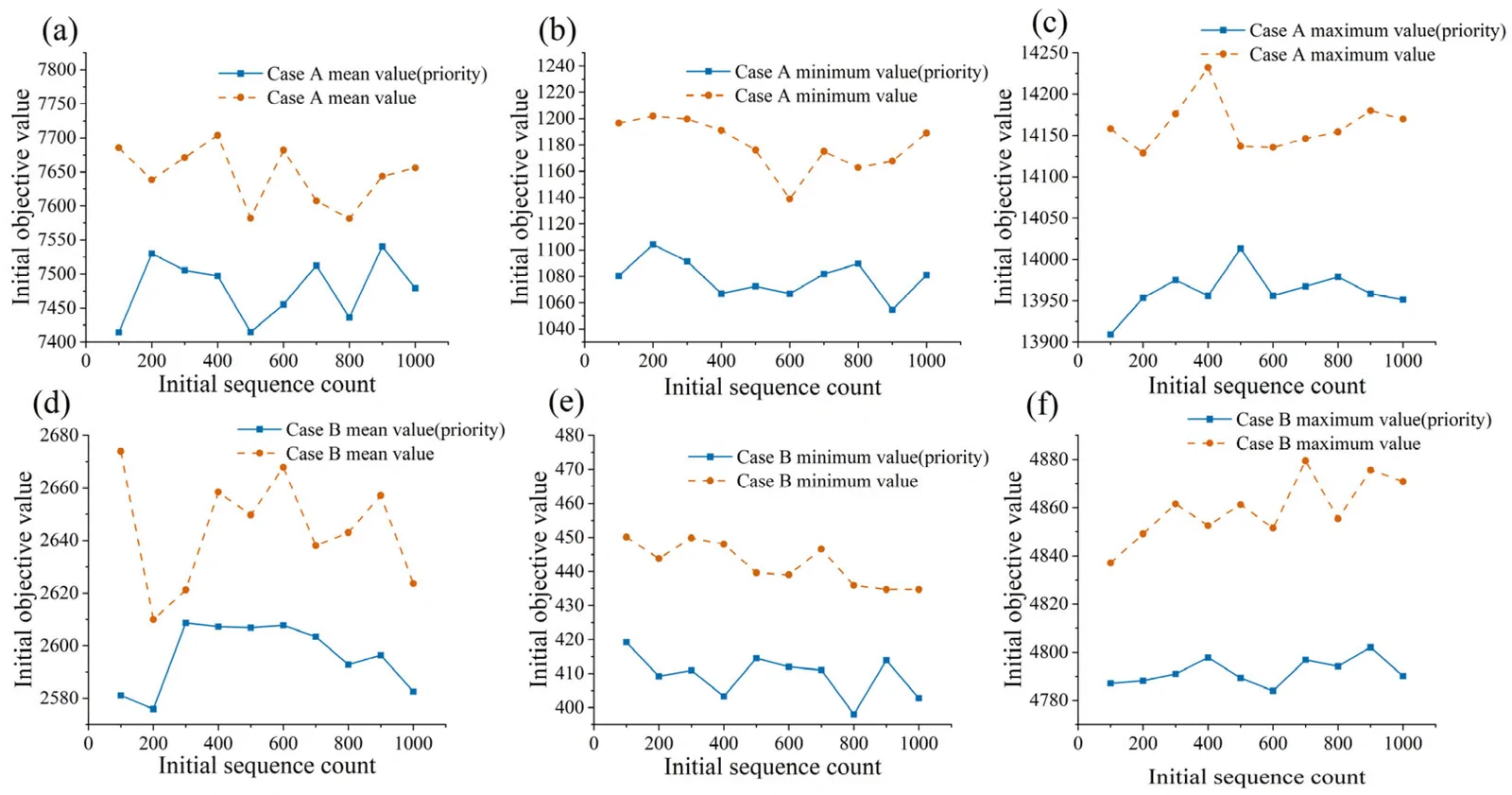
Comparison of initial objective values for Case Studies A and B. (**a**) Mean value for Case Study A; (**b**) minimum value for Case Study A; (**c**) maximum value for Case Study A; (**d**) mean value for Case Study B; (**e**) minimum value for Case Study B; (**f**) maximum value for Case Study B.

To validate the effectiveness of the improved interference matrix generation method for initial disassembly sequences, comparative experiments were conducted on two battery packs. The number of initial sequences was increased in intervals of 100, and all tests were performed under balanced mode. Each configuration was repeated 50 times to obtain average results, as shown in Figure 16.

Figure 16a,d display the mean initial objective values of disassembly sequences generated by the proposed priority-based improved interference matrix method and the conventional interference matrix method for Case Studies A and B, respectively. The values for both methods fluctuate with the initial sequence count without showing a clear linear pattern, while the proposed method consistently yields lower mean objective values. Figure 16b,e compare the minimum initial objective values, whereas Figure 16c,f compare the maximum initial objective values. Across these within-case comparisons, the proposed priority-based method consistently produces lower values than the conventional method, indicating an improvement in the baseline quality of the generated initial sequences. Initial objective values are calculated using unnormalised components to prevent normalisation from compressing interference penalties, thereby ensuring a consistent within-case comparison of the initialisation strategies.

#### 4.3.2. Comparison of Different Algorithms

To assess the performance of the proposed method quantitatively, comparative experiments were conducted against two established population-based metaheuristics, namely self-adaptive SSO [27] and teaching–learning-based optimisation (TLBO) [41], as well as the enhanced discrete Bees Algorithm (EDBA) for ablation analysis. SSO and TLBO were selected as representative swarm-based and learning-based optimisation methods, respectively, while EDBA was used as the ablation baseline by replacing the LinUCB-based adaptive operator selection with an equal-probability random operator-selection strategy, with all other optimisation components unchanged.

A 450-iteration budget was used for the population-size, iteration-budget, case-specific, and scalability analyses of LinUCB-BA. The separate comparative experiment used a common budget of 500 iterations without early stopping for all four algorithms. Each algorithm was executed for 50 independent trials per case study with a population size of 80.

**Figure 17:**
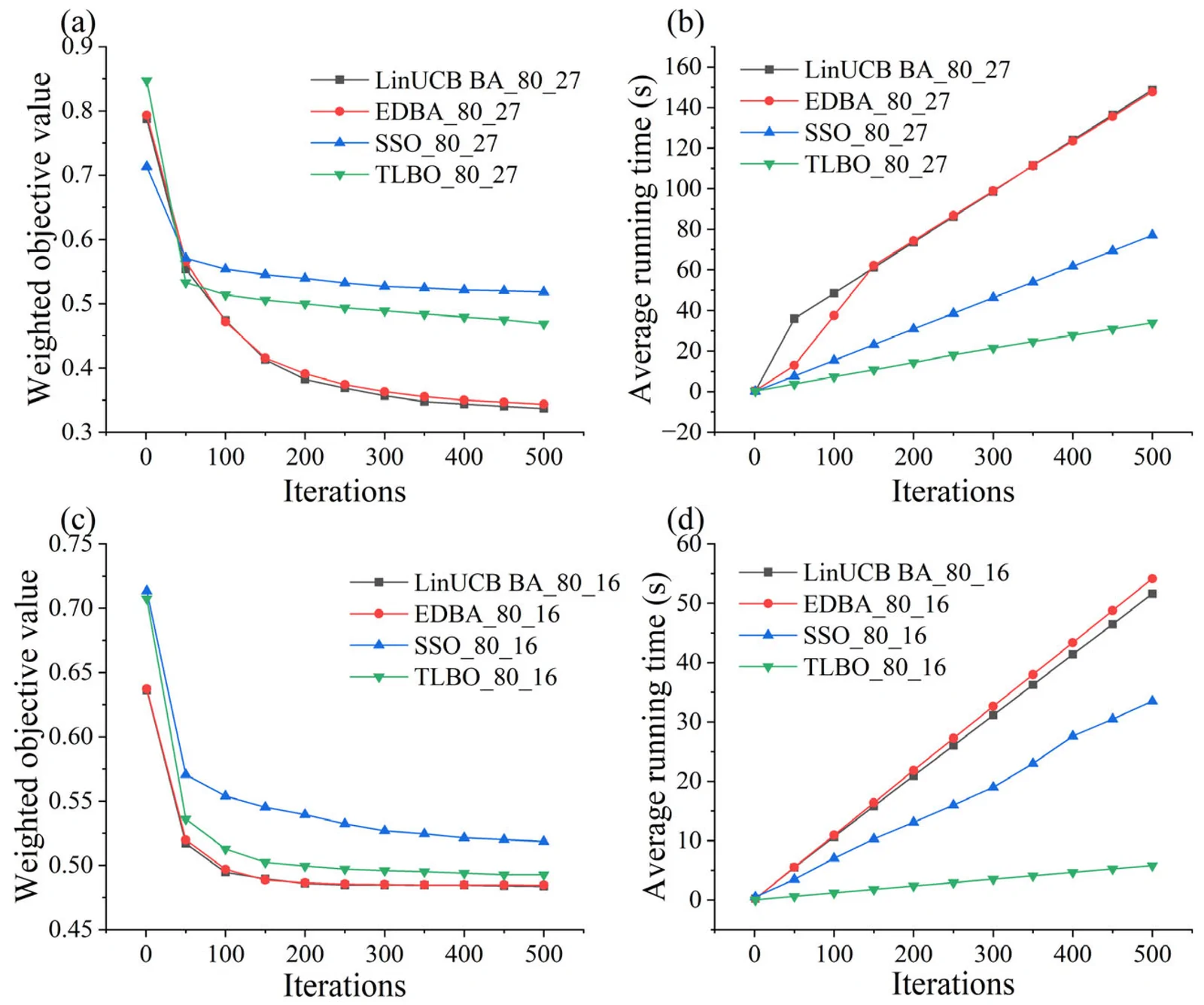
Optimisation performance and computational time comparison of different algorithms. (**a**) Convergence curves for Case A; (**b**) running time for Case A; (**c**) convergence curves for Case B; (**d**) running time for Case B.

Figure 17a,c present the convergence curves of the four algorithms for Case Studies A and B, respectively, while Figure 17b,d compare their average running times. As shown in Figure 17a,c, all four algorithms rapidly reduce the objective value during the initial iterations. SSO and TLBO converge quickly but show limited subsequent improvement, whereas both EDBA and LinUCB-BA continue to improve during the later search stages. Compared with EDBA, LinUCB-BA follows a similar early convergence trend and attains numerically lower objective values during the later iterations; however, as reported in Table 10, the pairwise differences are not statistically significant. As shown in Figure 17b,d, the computational time of LinUCB-BA is nearly identical to that of EDBA, indicating that adaptive operator selection introduces only negligible computational overhead relative to the EDBA ablation baseline.

The Wilcoxon signed-rank test was employed to evaluate the statistical significance of the pairwise performance differences between LinUCB-BA and each comparator in the two case studies. Since six pairwise comparisons were conducted, a Bonferroni correction was applied to the nominal significance level of α = 0.05, resulting in an adjusted threshold of α′ = 0.00833. As reported in Table 10, LinUCB-BA differs significantly from SSO and TLBO in both case studies (all *p* < 0.00833), whereas the differences from EDBA are not statistically significant in Case A (*p* = 0.2507) or Case B (*p* = 0.7030).

Table 10 summarises the best-found objective value, mean objective value, and standard deviation obtained by the four algorithms over 50 independent runs. LinUCB-BA achieved the lowest mean normalised weighted objective value in both case studies. In Case A, the mean objective value obtained by LinUCB-BA was 0.3369, compared with 0.3437 for EDBA, 0.4687 for TLBO, and 0.5524 for SSO, corresponding to reductions of approximately 1.98%, 28.12%, and 39.01%, respectively. In Case B, LinUCB-BA achieved a mean objective value of 0.4812, corresponding to reductions of approximately 0.43%, 2.33%, and 7.19% relative to EDBA, TLBO, and SSO, respectively. These results show clear numerical improvements over all three comparators. 

Regarding run-to-run variability, LinUCB-BA has numerically lower standard deviations than EDBA in both Case A (0.0171 versus 0.0180) and Case B (0.0063 versus 0.0078), indicating a small descriptive improvement in run-to-run variability that, as previously mentioned, lacks statistical significance. Notably, both algorithms reach the same best-found objective value of 0.4766 in Case B. Taken together, these results suggest that adaptive operator selection may provide modest numerical gains, particularly in the larger Case A, but the current trials do not establish a statistically significant advantage over equal-probability operator selection.

#### 4.3.3. Scalability Analysis

**Figure 18:**
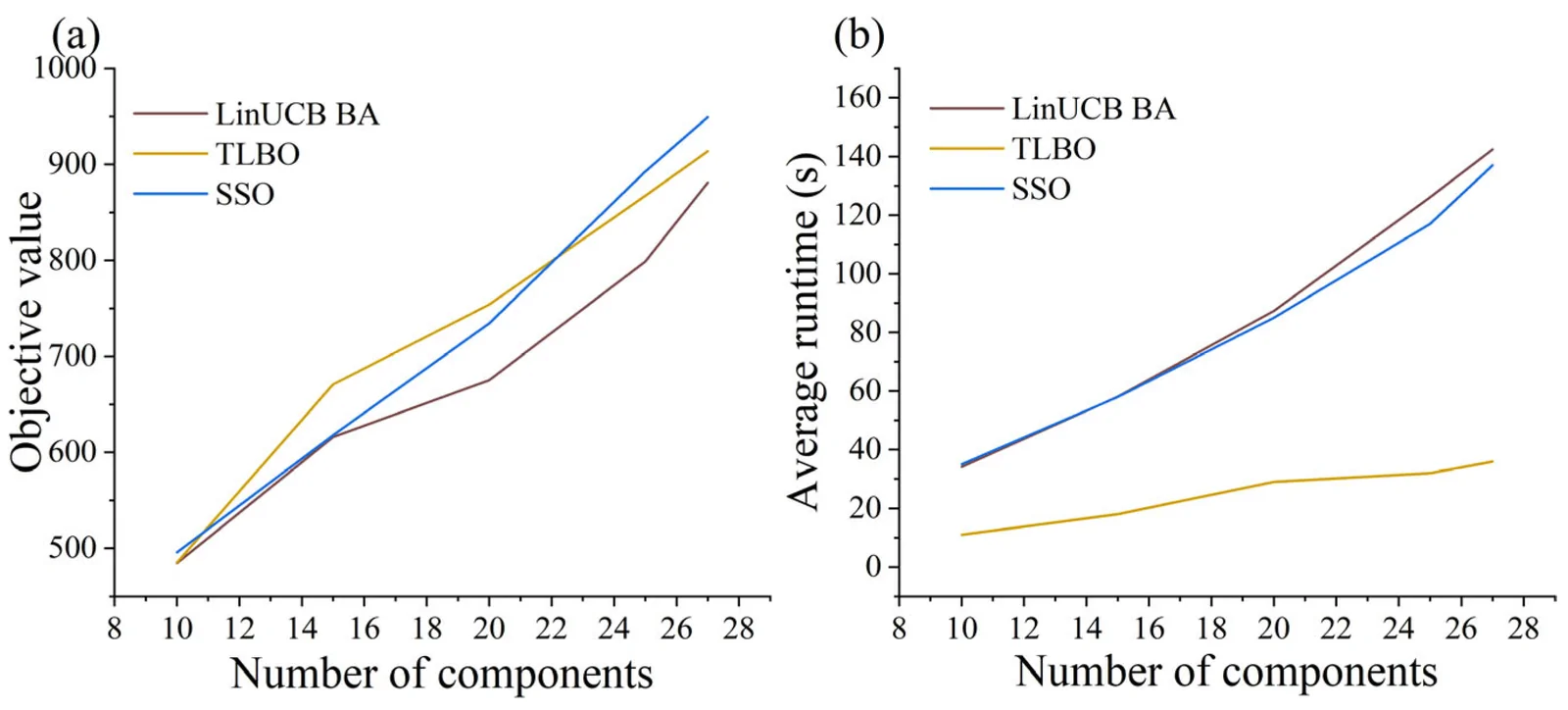
Scalability analysis of LinUCB-BA, TLBO, and SSO. (**a**) Best objective values calculated from unnormalised objective components. (**b**) Average runtime comparison.

To evaluate performance across different problem sizes, an additional scalability experiment was conducted using Case Study A, which contains more components and is therefore suitable for constructing problems of different sizes. The population size was set to 80, and the maximum number of iterations was set to 450. Subsets consisting of N = 10, 15, 20, and 25 components were generated from Case Study A and evaluated alongside the full 27-component problem. Consistent with Section 4.3.1, Figure 18a reports unnormalised best objective values to avoid introducing size-specific normalisation intervals. Consequently, objective values are only compared within identical problem sizes, while cross-size scalability is assessed via average runtime (Figure 18b).

## 5. Discussion

To address the challenges of complex constraint handling, combinatorial explosion, and premature convergence in the disassembly sequence planning (DSP) of end-of-life (EoL) EV batteries, this study proposed a disassembly sequence optimisation approach that combines a process-oriented hard-constraint rule base, a LinUCB learning mechanism, and an enhanced BA. The method integrates constraint-aware sequence generation with adaptive search, thereby improving both solution feasibility and optimisation performance. It consistently outperformed the benchmark SSO and TLBO algorithms across two representative case studies.

The adaptive operator-selection mechanism introduces additional computational overhead. For a 27-component problem (population 80, 500 iterations), LinUCB-BA required 125 s per run, compared with 34 s for TLBO and 77 s for SSO. This additional effort is accompanied by significant optimisation improvements relative to SSO and TLBO: LinUCB-BA reduced the mean normalised weighted objective value by 39.01% and 28.12% in Case Study A, and by 7.19% and 2.33% in Case Study B, respectively. These improvements are statistically significant under the Bonferroni-corrected threshold.

Comparisons were conducted using identical population sizes and iteration numbers (a consistent nominal search budget), demonstrating improved solution quality rather than superiority under a fixed wall-clock time limit. Such time-constrained comparisons remain a direction for future work. Furthermore, scalability tests (10 to 27 components) showed that LinUCB-BA yields the lowest objective value for larger instances, albeit with higher runtimes than the benchmarks.

A key performance driver is the constraint-handling framework. Embedding a process-oriented hard-constraint rule base within the interference matrix ensures candidate sequences satisfy key safety and process constraints during generation, eliminating invalid sequences before optimisation. Consequently, initial sequences generated by this method demonstrated consistently lower objective values than those using conventional matrices.

Ablation testing against the EDBA isolated the effect of the LinUCB adaptive selection. While LinUCB-BA achieved numerical reductions in the mean objective value (1.98% in Case A; 0.43% in Case B) and slightly lower run-to-run variability, these differences were not statistically significant. Therefore, the robust advantage over SSO and TLBO stems from the entire integrated framework—constraint handling, feasible sequence generation, and adaptive search—rather than the adaptive operator selection alone.

A probable explanation for the different improvement magnitudes is the difference in problem complexity. The 27-component case has a larger and more constrained search space, potentially providing more opportunities for the benefits of constraint-guided search to emerge. In contrast, the smaller search space of the 16-component case may leave less room for further improvement. This interpretation is plausible but cannot be regarded as a proven causal mechanism based on the two cases alone.

Contextually, previous matrix-based models efficiently handled geometric relationships but neglected process-level constraints, while knowledge graphs and Petri nets captured complex relationships but resisted direct integration into heuristic searches. This study bridges that gap by embedding process constraints directly into the interference matrix. Furthermore, unlike existing studies that merge reinforcement learning and metaheuristics using value-based mechanisms, this approach employs contextual-bandit-based operator selection, explicitly conditioning decisions on the current search state.

Another practical advantage of the method is its flexibility to accommodate diverse operational priorities through four distinct decision modes: balanced, economic, efficient, and ergonomic. Because the framework employs a weighted-sum multi-criteria formulation rather than a Pareto-based evolutionary approach, it generates a single, preference-specific solution for a given scenario. Exploring Pareto-based methods, such as the non-dominated sorting genetic algorithm II (NSGA-II) and the non-dominated sorting genetic algorithm III (NSGA-III), to generate multiple non-dominated plans remains a valuable avenue for future research.

Despite its advantages, the framework has limitations. It assumes deterministic task execution at the planning level, ignoring practical interaction variables such as part-transfer delays or human execution variability. It also focuses on single-workstation collaborative scenarios, lacking support for multi-station or parallel disassembly. Finally, it assumes fixed topology and task parameters. The parameter-sensitivity analysis was limited to the LinUCB exploration coefficient, population size, and iteration budget. The numbers of elite and selected sites, the allocation of employed bees, and the other fixed algorithm parameters were not independently varied. Therefore, the present results should not be interpreted as demonstrating insensitivity to all BA parameters. Future work will investigate condition-aware planning, incorporating battery state-of-health, residual lifetime, and sensor-derived disturbance data to support dynamic, real-time safety assessments and sequence replanning.

## 6. Conclusions

This paper proposes a constraint-guided adaptive optimisation framework for disassembly sequence optimisation, where a process-oriented hard-constraint rule base and LinUCB-based operator selection are incorporated into the BA framework. The framework integrates process-level constraint handling with context-aware adaptive search, enabling the optimisation process to focus on physically feasible solutions while dynamically selecting suitable search operators.

The experimental results demonstrate that the proposed LinUCB-BA method achieves significantly lower weighted objective values than SSO and TLBO across the two battery-pack cases. The ablation comparison with EDBA yields slightly lower mean objective values, but the differences are not statistically significant, suggesting a potential rather than confirmed advantage of adaptive operator selection under the current experimental settings. The scalability analysis also shows that the proposed method remains applicable as the number of disassembly components increases, although the computational cost increases with problem size.

The novelty of this work does not lie simply in combining the BA with reinforcement learning. Rather, it lies in integrating process-oriented hard constraints directly into feasible sequence generation and coupling them with context-aware LinUCB-based operator selection within the BA framework. The hard-constraint rule base incorporates topological relationships, process constraints, and component precedence rules into the improved interference matrix, while LinUCB dynamically selects search operators according to the observed optimisation state. This integration provides a constraint-guided and adaptive search mechanism for complex EoL-EV battery disassembly sequence optimisation.

Furthermore, the proposed method provides decision-support capabilities for human–robot collaborative battery disassembly by generating feasible and cost-effective disassembly strategies under complex process constraints, thereby contributing to more efficient and sustainable battery recycling operations.

Future research will focus on extending the framework to dynamic and uncertain disassembly environments by incorporating sensor-derived disturbance information, execution variability, tool failures, and changing human–robot task requirements. Battery health and degradation information will also be considered to support condition-aware safety assessment, task allocation, and sequence replanning.

## Figures and Tables

**Table 1:** Hazard level of components.

Component Attributes	Hazard Level
Fasteners (bolts/screws)	0.2
Enclosures (shells/covers)	0.4
Electrical components (bus bars, BMS, etc.)	0.6
Core energy storage (battery modules)	0.9

**Table 2:** Algorithm parameter definitions.

Symbol	Definition
$n_{s}$	Initial population size of scout bees
iter	Total number of iterations of the algorithm
$m_{t}$	Total number of scout bees in the foraging population
$n_{e}$	Number of elite sites selected per iteration
$n_{b}$	Number of non-elite best sites selected per iteration
$n_{re}$	Number of employed bees allocated to each elite site
$n_{rb}$	Number of employed bees allocated to each non-elite best site

**Table 3:** Data on Tesla battery disassembly.

No.	Component (Number)	Disassembly Point (mm)	Method 1 (s)	Method 2 (s)	Method 3 (s)	Method 4 (s)
1	Housing upper part bolts (68)	[1375, 767.5, 130]	748	544	680	612
2	Upper shell protruding cover	[2562.5, 792.5, 250]	10,000	51	10,000	52
3	Upper shell body cover	[1537.5, 792.5, 150]	10,000	63	10,000	64
4	Main fuse	[2562.5, 430, 120]	15	12	14	13
5	Cooling system	[2562.5, 1155, 240]	10,000	123	10,000	124
6	Fire-proof plate A	[2562.5, 792.5, 250]	8	5	7	6
7	Fire-proof plates (14)	[1537.5, 767.5, 130]	112	70	98	84
8	Bolt protection cover (24)	[1537.5, 767.5, 125]	336	264	312	288
9	Battery module bolts (28)	[1537.5, 767.5, 115]	280	196	252	224
10	Battery module A	[2562.5, 792.5, 240]	21	18	20	19
11	Battery module B	[2562.5, 792.5, 120]	21	18	20	19
12	Battery module C	[2250, 1155, 120]	21	18	20	19
13	Battery module D	[2250, 430, 120]	21	18	20	19
14	Battery module E	[1925, 1155, 120]	21	18	20	19
15	Battery module F	[1925, 430, 120]	21	18	20	19
16	Battery module G	[160, 1155, 120]	21	18	20	19
17	Battery module H	[160, 430, 120]	21	18	20	19
18	Battery module I	[1275, 115.5, 120]	21	18	20	19
19	Battery module J	[1275, 430, 120]	21	18	20	19
20	Battery module K	[950, 1155, 120]	21	18	20	19
21	Battery module L	[950, 430, 120]	21	18	20	19
22	Battery module M	[625, 1155, 120]	21	18	20	19
23	Battery module N	[625, 430, 120]	21	18	20	19
24	Battery module O	[300, 1155, 120]	21	18	20	19
25	Battery module P	[300, 430, 120]	21	18	20	19
26	Electrical part	[750, 767.5, 120]	20	17	19	18
27	Housing under part	[1375, 767.5, 0]	38	23	37	24

**Table 4:** Weight coefficients for four disassembly optimisation modes.

Mode	Time Cost	Payment Cost	Utility Value
Balance mode	0.33	0.33	0.33
Economic mode	0.25	0.50	0.25
Efficient mode	0.50	0.25	0.25
Ergonomic mode	0.25	0.25	0.50

**Table 5:** Disassembly data for the Mercedes-Benz battery pack.

No.	Component	Disassembly Point (mm)	Method 1 (s)	Method 2 (s)	Method 3 (s)	Method 4 (s)
1	Housing upper part	[522.5, 372.5, 224]	798	582	725	655
2	Upper shell protruding cover	[522.5, 0, 224]	10,004	15	10,003	16
3	Copper busbar A	[40, 310, 204]	14	11	13	12
4	Copper busbar B	[685, 310, 204]	14	11	13	12
5	Copper busbar C	[685, 310, 204]	14	11	13	12
6	Copper busbar D	[40, 750, 204]	14	11	13	12
7	Copper busbar E	[40, 526, 124]	16	13	15	14
8	Copper busbar F	[320, 80, 199]	12	9	11	10
9	Battery filter	[470, 35, 199]	20	17	19	18
10	Main fuse	[240, 70, 134]	15	12	14	13
11	Copper busbar connector	[215, 40, 180]	19	16	18	17
12	Desiccant box	[230, 1000, 199]	12	9	11	10
13	Battery Management System	[530, 1010, 206]	16	13	15	14
14	Battery module A	[350, 310, 184]	21	18	20	19
15	Battery module B	[350, 750, 184]	21	18	20	19
16	Housing under part	[522.5, 0, 210]	10,000	15	10,000	16

**Table 6:** Statistical results of engineering performance metrics over 50 independent runs in Case Study A.

Metric	Mean ± Std
Time (s)	1951.73 ± 34.39
Cost ($)	688.26 ± 19.65
Utility (%)	85.70 ± 0.32

**Table 7:** Best-found results under four disassembly modes in Case Study A.

Type	Best-Found Solution	Value	Time Cost (s)	Payment Cost ($)	Utility Value (%)	Weighted Objective Value
Balance mode	Sequence	1-2-6-5-3-7-8-9-19-17-15-13-4-10-11-12-14-16-18-20-22-24-26-21-23-25-27	1913.24	668.95	85.92	0.3121
	Direction	5-5-5-5-5-5-5-5-5-5-5-5-5-4-5-5-5-5-5-5-5-5-5-5-5-5-5				
	Resource	1-2-2-2-2-1-1-1-1-1-1-1-1-1-1-1-1-1-1-1-1-1-1-1-1-1-2				
Economic mode	Sequence	1-2-6-5-3-7-8-9-19-17-15-13-4-10-11-12-14-16-18-20-22-24-26-21-23-25-27	1911.24	668.39	85.92	0.2466
	Direction	5-5-5-5-5-5-5-5-5-5-5-5-5-4-5-5-5-5-5-5-5-5-5-5-5-5-5				
	Resource	1-2-2-2-2-1-1-1-1-1-1-1-1-1-1-1-1-1-1-1-1-1-1-1-1-1-2				
Efficient mode	Sequence	1-2-6-5-3-26-7-8-9-16-14-12-24-22-10-20-11-18-4-13-15-17-19-21-23-25-27	1642.03	1017.60	78.85	0.4576
	Direction	5-5-5-5-5-5-5-5-5-5-5-5-5-5-5-5-5-5-5-5-5-5-5-5-5-5-5				
	Resource	2-2-2-2-2-1-2-2-2-2-2-2-1-1-2-1-2-1-2-2-2-2-1-1-1-1-2				
Ergonomic mode	Sequence	1-2-6-5-3-7-8-9-16-14-12-10-11-4-13-15-17-19-21-26-23-25-24-22-20-18-27	1918.30	671.23	85.92	0.3084
	Direction	5-5-5-5-3-5-5-5-5-5-5-5-5-5-5-5-5-5-5-5-5-5-5-5-5-5-5				
	Resource	1-2-2-2-2-1-1-1-1-1-1-1-1-1-1-1-1-1-1-1-1-1-1-1-1-1-2				

**Table 8:** Statistical results of engineering performance metrics over 50 independent runs in Case Study B.

Metric	Mean ± Std
Time (s)	728.79 ± 4.60
Cost ($)	393.02 ± 11.19
Utility (%)	79.85 ± 0.24

**Table 9:** Best-found results under four disassembly modes in Case Study B.

Type	Best-Found Solution	Value	Time Cost (s)	Payment Cost ($)	Utility Value (%)	Weighted Objective Value
Balance mode	Sequence	1-2-5-4-9-8-10-11-3-7-6-12-13-15-14-16	725.11	383.12	79.67	0.5242
	Direction	5-5-5-5-5-5-5-5-5-5-5-5-5-5-5-5				
	Resource	2-2-1-1-1-1-1-1-1-1-1-1-1-1-1-2				
Economic mode	Sequence	1-2-9-8-10-5-11-3-7-6-12-13-4-14-15-16	865.75	250.78	80.74	0.4069
	Direction	5-5-5-5-5-5-5-5-5-5-5-5-5-5-5-5				
	Resource	2-2-2-2-2-1-2-2-2-2-2-2-1-1-2-1				
Efficient mode	Sequence	1-2-5-9-8-10-11-3-7-6-12-13-4-14-15-16	681.42	447.41	79.02	0.3986
	Direction	5-5-5-5-5-5-5-5-5-5-5-5-5-5-5-5				
	Resource	2-2-1-2-2-2-2-2-2-2-2-2-1-1-2-1				
Ergonomic mode	Sequence	1-2-4-5-9-8-10-11-3-7-6-12-13-15-14-16	887.53	316.36	82.61	0.4526
	Direction	5-5-5-5-5-5-5-5-5-5-5-5-5-5-5-5				
	Resource	1-2-1-1-2-3-2-2-3-3-3-2-2-1-1-1				

**Table 10:** Statistical comparison of optimisation performance among different algorithms.

Algorithm	Case Study	Best	Mean ± Std	*p*-Value vs. LinUCB-BA
TLBO	A	0.4040	0.4687 ± 0.0177	1.78 × 10^−15^
SSO	A	0.5105	0.5524 ± 0.0206	1.78 × 10^−15^
EDBA	A	0.3167	0.3437 ± 0.0180	0.2507
LinUCB-BA	A	0.3121	0.3369 ± 0.0171	—
TLBO	B	0.4766	0.4927 ± 0.0092	8.01 × 10^−6^
SSO	B	0.4850	0.5185 ± 0.0160	2.39 × 10^−9^
EDBA	B	0.4766	0.4833 ± 0.0078	0.7030
LinUCB-BA	B	0.4766	0.4812 ± 0.0063	—

Std: Standard deviation.

## Data Availability

The raw data supporting the conclusions of this article are available on request from the corresponding author.

## Abbreviations

The following abbreviations are used in this manuscript:

BA	Bees Algorithm
EDBA	Enhanced Discrete Bees Algorithm
EoL	End of Life
EV	Electric Vehicle
DSP	Disassembly Sequence Planning
ILP	Integer Linear Programming
LCD	Liquid Crystal Display
LinUCB	Linear Upper Confidence Bound
MOEA/D	Multi-Objective Evolutionary Algorithm Based on Decomposition
SSO	Simplified Swarm Optimisation
TLBO	Teaching–Learning-Based Optimisation
WEEE	Waste Electrical and Electronic Equipment
